# Publisher Correction: The kinetics of nsp7-11 polyprotein processing and impact on complexation with nsp16 among human coronaviruses

**DOI:** 10.1038/s41467-025-64890-1

**Published:** 2025-10-09

**Authors:** Kira Schamoni-Kast, Boris Krichel, Tomislav Damjanović, Fatema-Aqila Said, Thomas Kierspel, Sibel Toker, Charlotte Uetrecht

**Affiliations:** 1 CSSB Centre for Structural Systems Biology, Deutsches Elektronen Synchroton DESY, Leibniz Institute of Virology, University of Lübeck, Hamburg, Germany; 2 Institute of Chemistry and Metabolomics, University of Lübeck, Lübeck, Germany

**Keywords:** SARS-CoV-2, Kinetics, Structural biology, Proteases

Correction to: *Nature Communications* 10.1038/s41467-025-61554-y, published online 09 September 2025

In the version of the article initially published, the values in Fig. 4B were incorrect, and the left-hand column should have shown the units: “*k* [min^−1^]”. For reference, the original, uncorrected figure can be found in the Supplementary Information accompanying this notice. The figure has been corrected in the HTML and PDF versions of the article.

## Supplementary information

Original, uncorrected Fig. 4.

