# Supplementary material for: The kinetics of nsp7-11 polyprotein processing and impact on complexation with nsp16 among human coronaviruses
Source: Nat Commun. 2025 Sep 9;16:8244. doi: 10.1038/s41467-025-61554-y (PMC12420816; doi:10.1038/s41467-025-61554-y)
Supplement: Supplementary file 1 — Supplementary Information [file 41467_2025_61554_MOESM1_ESM.pdf]

The kinetics of nsp7-11 polyprotein processing and impact on complexation with nsp16 in human coronaviruses.

Kira Schamoni-Kast[1,2†] Boris Krichel[1,2†], Tomislav Damjanović[1,2], Fatema-Aqila Said[1], Thomas Kierspel[1], Sibel Toker[3], Charlotte Uetrecht[1,2*]

[1]CSSB Centre for Structural Systems Biology, Deutsches Elektronen-Synchrotron DESY & Leibniz Institute of Virology (LIV) & University of Lübeck, Notkestraße 85, 22607 Hamburg, Germany.

[2] Institute of Chemistry and Metabolomics, University of Lübeck, Ratzeburger Allee 160, 23562 Lübeck, Germany

[3]Leibniz Institute of Virology, Martinistraße 52, 20251 Hamburg, Germany

†These authors contributed equally to this work

*Correspondence: charlotte.uetrecht@cssb-hamburg.de

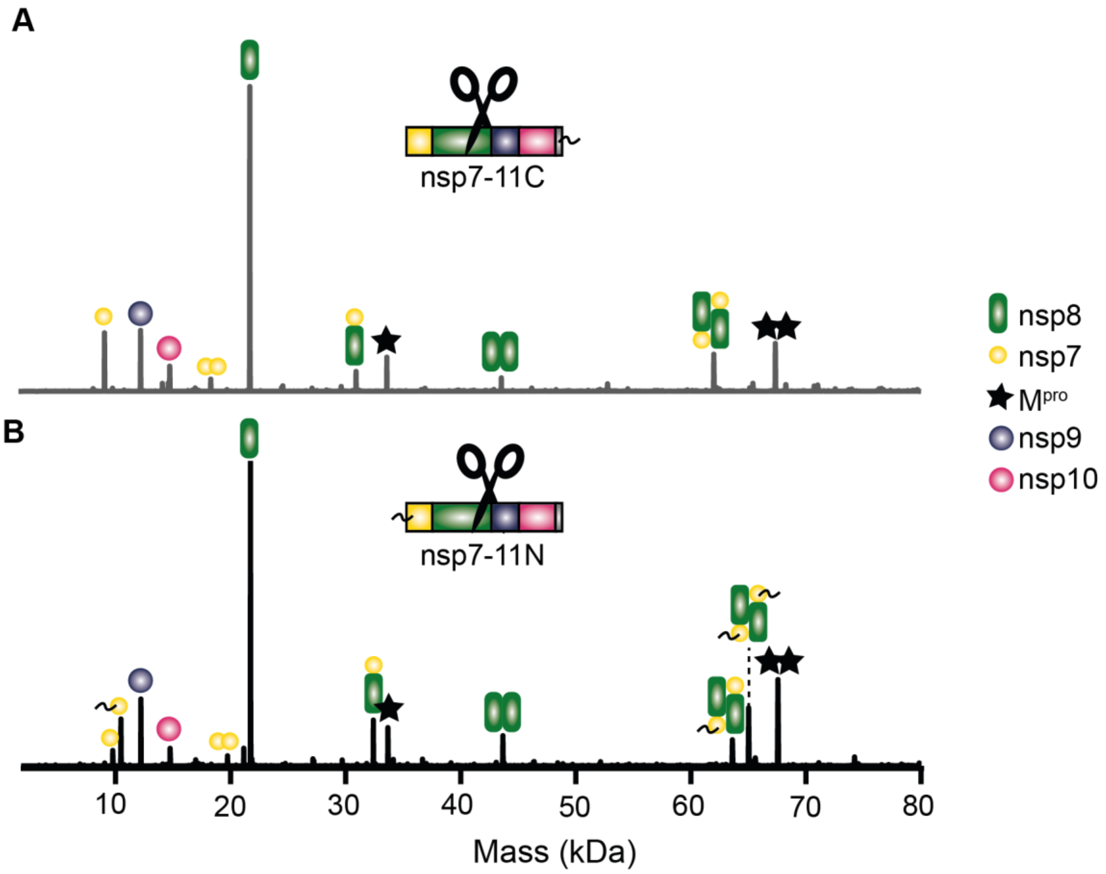

Supplementary Figure S1: Complete processing of nsp7-11C and nsp7-11N. M^{pro} (3 µM) was mixed with 18 µM nsp7-11C (**A**) or nsp7-11N (**B**) and incubated for 20 h at 4˚C. Detected mass species and complexes are shown as deconvoluted spectra. Source data are provided as a Source Data file.

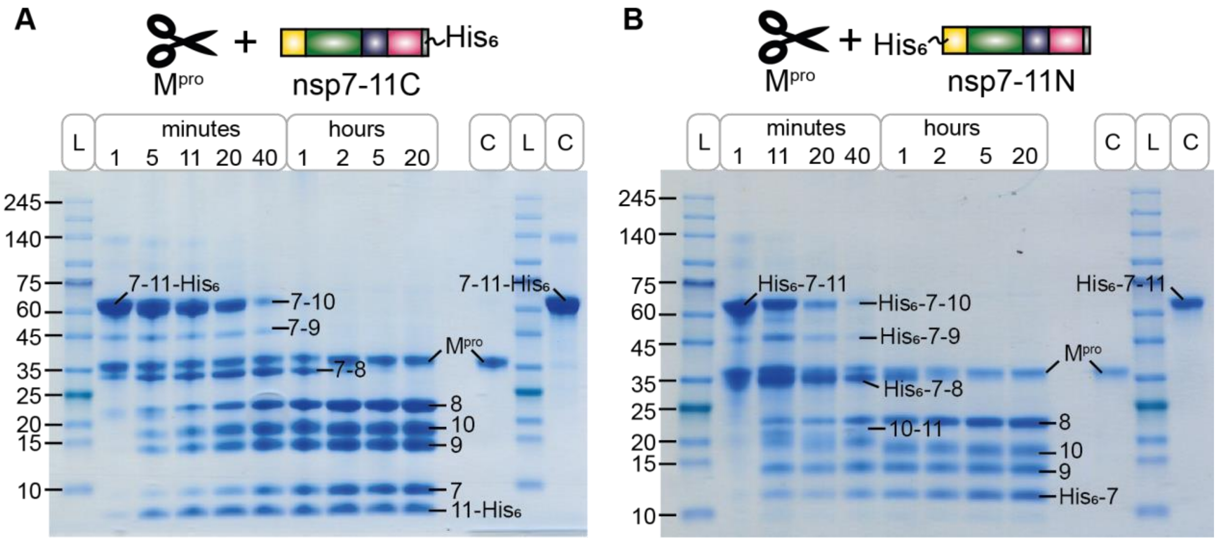

Supplementary Figure S2: SDS-PAGE of nsp7-11C processing (A) and nsp7-11N processing (B) showing protein marker ladder (L) and controls (C) of M^{pro} and nsp7-11C or nsp7-11N. Both constructs nsp7-11C and nsp7-11N were mixed at 36 µM with 14 µM M^{pro} and incubated at 4°C. Aliquots were withdrawn at indicated time points. Source data are provided as a Source Data file.

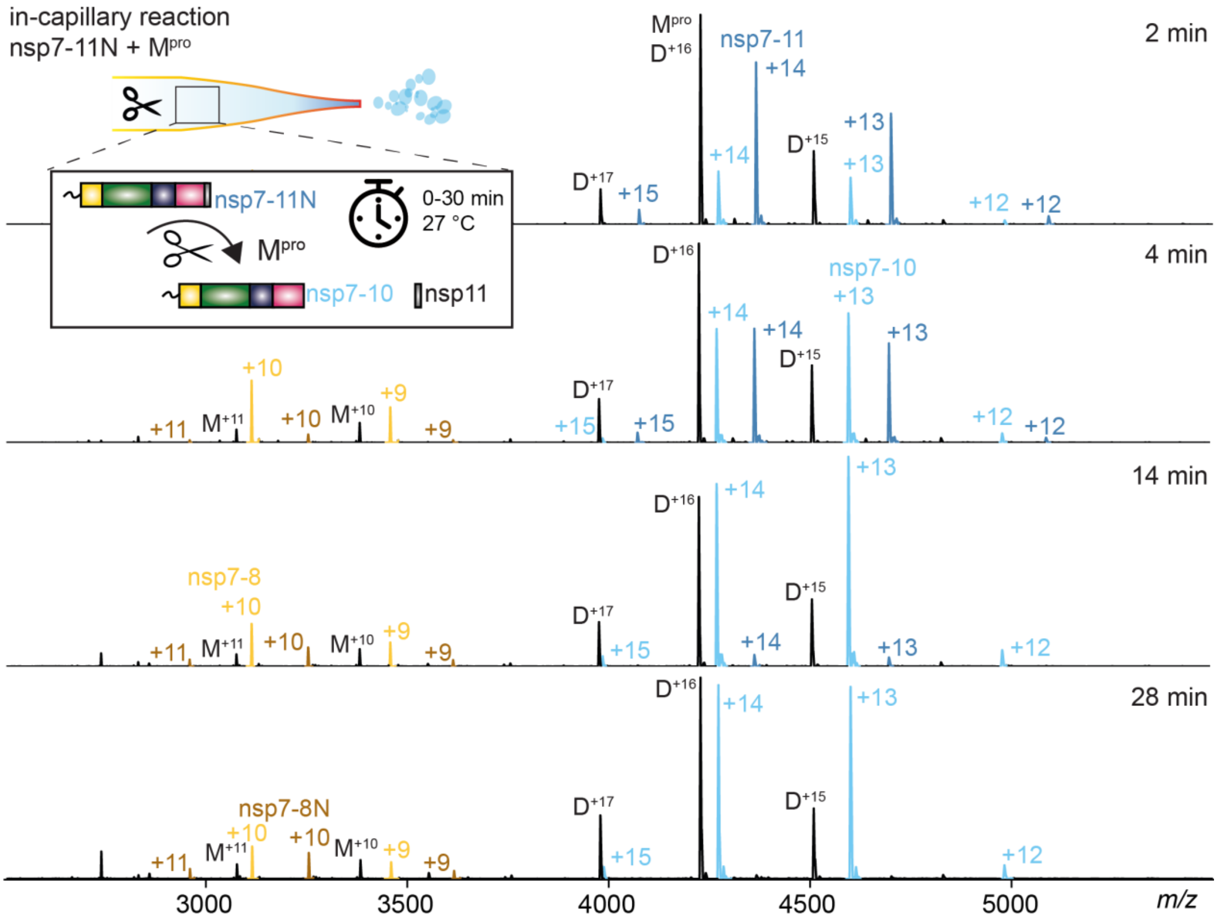

Supplementary Figure S3: Continuous processing of nsp7-11N (18 µM) with M^{pro} (3 µM) at 27°C. Representative mass spectra of nsp7-11N (18 µM) showing continuous in-capillary processing at 2 min, 4 min, 14 min and 28 min. Continuous processing of nsp7-11N reaches its crossing point at ~4 min, in which nsp7-10 (light blue) reaches similar intensities as nsp7-11 (dark blue) and then nsp7-10 becomes the predominant species (14 min, 28 min). M^{pro} dimer (D) peaks are shown in black. nsp7-8 (yellow) and nsp7-8N (brown) become populated over time. Source data are provided as a Source Data file.

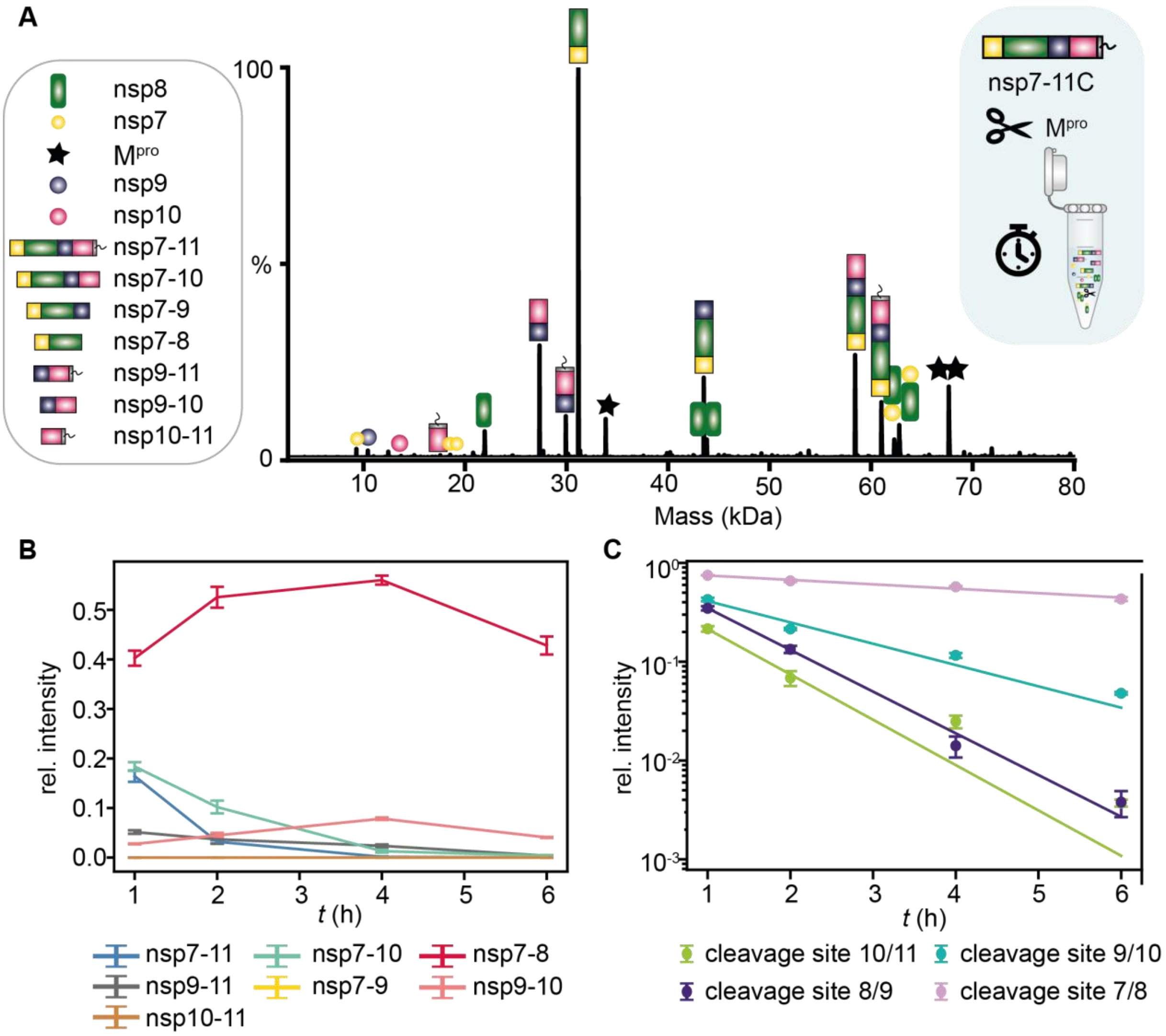

Supplementary Figure S4: Discrete processing approach of nsp7-11C (20 µM) and M^{pro} (10 µM) at 0°C (on ice). (**A**) A representative deconvoluted mass spectrum after 2 h. (**B**) Course of all deconvoluted intermediate species of the sub-polyprotein at indicated time points. Data points are connected for better visibility. (**C**) Determination of the rate constants *k* by following the depletion of the substrates corresponding to each cleavage site. The decay is represented as fitted line. After 24 h, all cleavage sites are processed and hence the data points, which are devoid of the plotted species, omitted. Mean and standard error, depicted as error bars, was calculated from triplicate measurements. Polyprotein processing of nsp7-11C was repeated three times with technical triplicates. Source data are provided as a Source Data file.

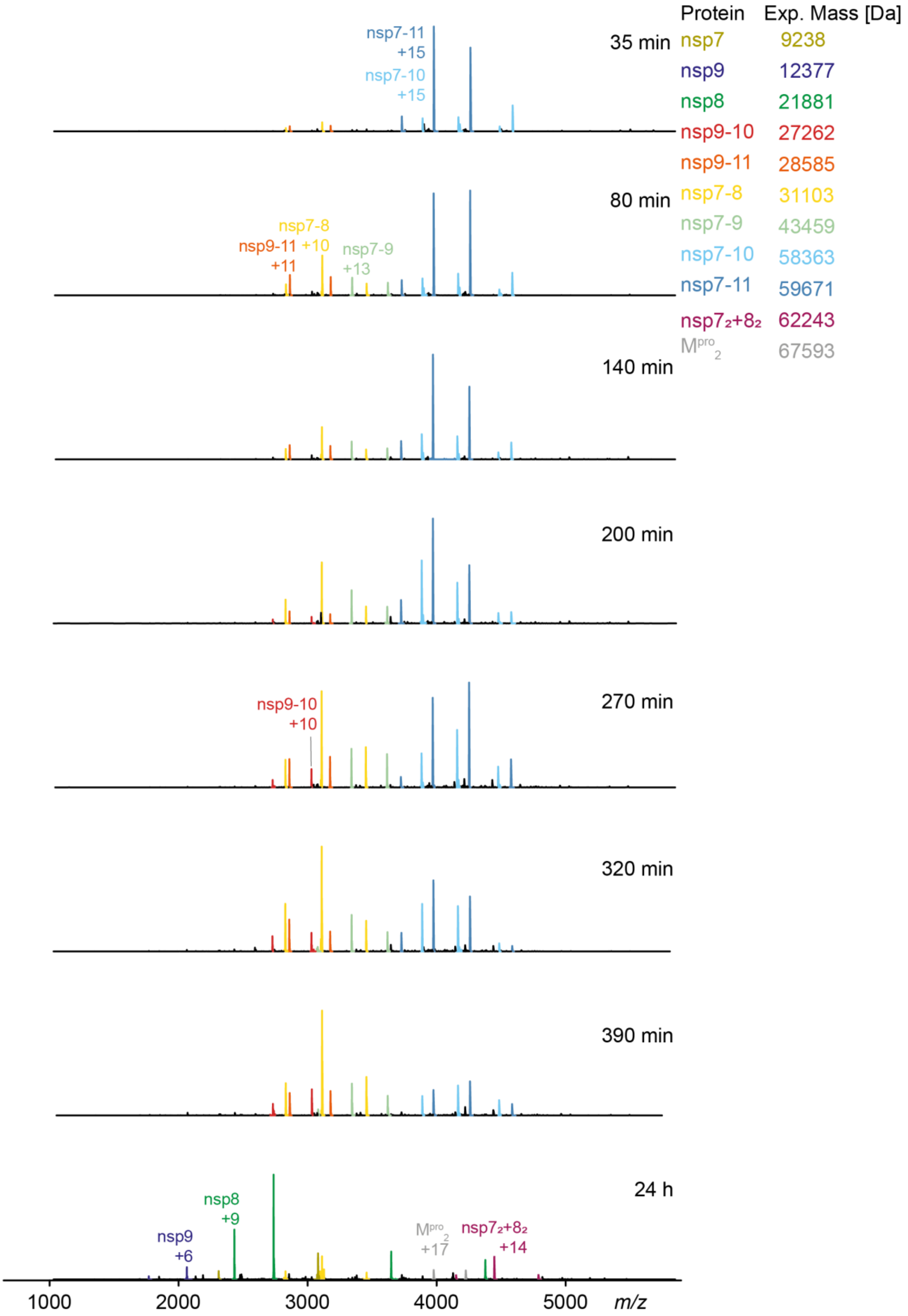

Supplementary Figure S5: Discontinuous processing of SARS-CoV-2 nsp7-11. Representative mass spectra from the time-resolved processing (35-390 min) of 19 µM nsp7-11 with 3.5 µM M^{pro} on ice (0˚C) are shown. Intermediate species nsp9-10 (red), nsp9-11 (brick red), nsp7-8 (yellow), nsp7-9 (cyan), nsp7-10 (light blue), nsp7-11 (steel blue) are colored. End point at 24 h (incubation at 4˚C) shows mature nsp7, nsp8, nsp9 and nsp7/nsp8 complexes (purple). Polyprotein processing of SARS-CoV-2 nsp7-11 with authentic termini was repeated twice with technical triplicates. Source data are provided as a Source Data file.

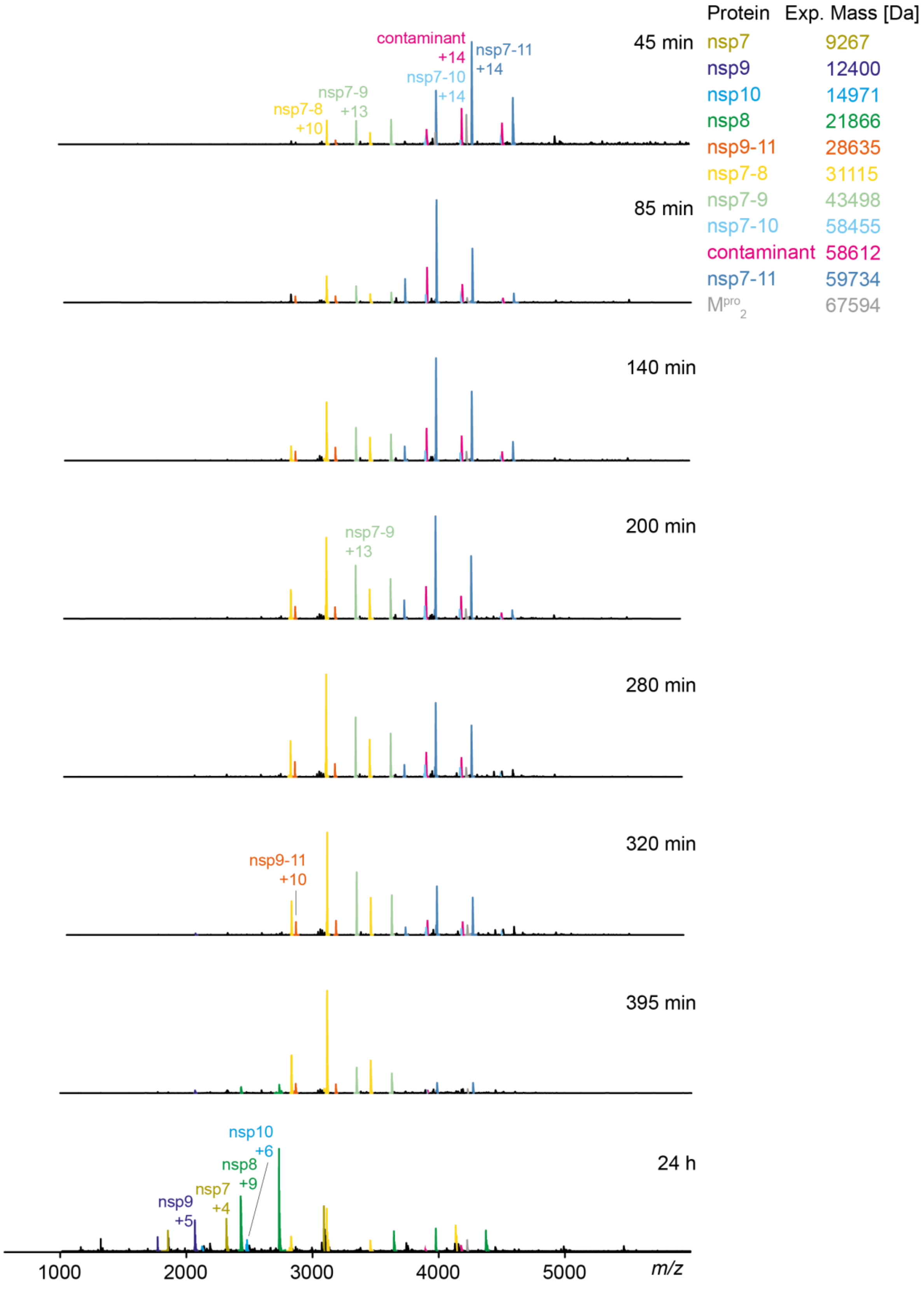

Supplementary Figure S6: Discontinuous processing of SARS-CoV nsp7-11. Representative mass spectra from the time-resolved processing (45-395 min) of 19 µM nsp7-11 with 3.5 µM M^{pro} on ice (0˚C) are shown. Intermediate species nsp9-11 (brick red), nsp7-8 (yellow), nsp7-9 (cyan), nsp7-10 (light blue), nsp7-11 (steel blue) are colored. The end point at 24 h (incubation at 4˚C) shows mature nsp7 (yellow), nsp8 (green), nsp9 (purple), nsp10 (blue). Heterotetramer $nsp7_2$+8_2 was detected in low intensities, and thus not visible in the shown spectra. Polyprotein processing of SARS-CoV nsp7-11 with authentic termini was repeated twice with technical triplicates. Source data are provided as a Source Data file.

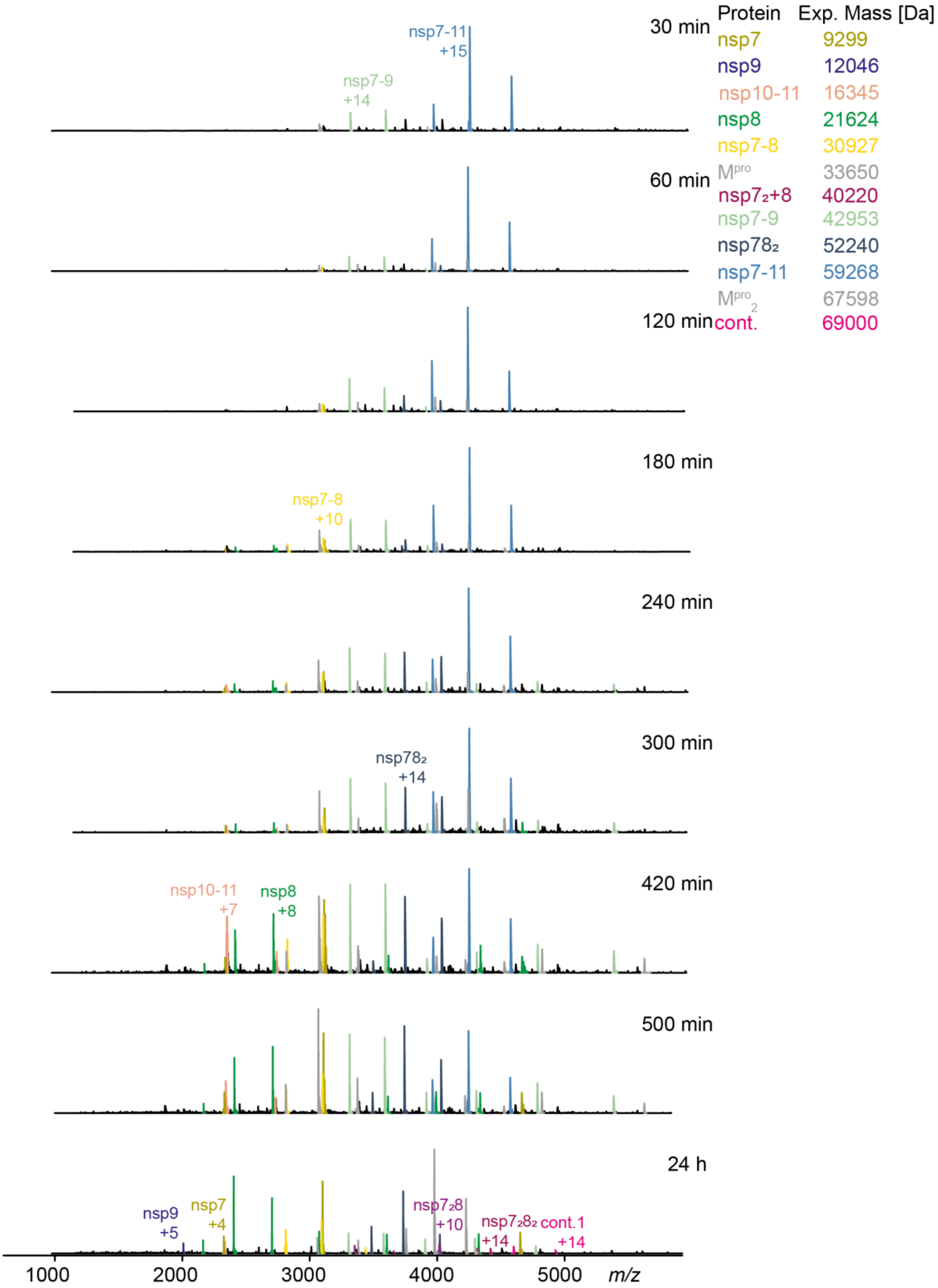

Supplementary Figure S7: Discontinuous processing of HCoV-229E nsp7-11. Representative mass spectra from the time-resolved processing (30-500 min) of 17 µM nsp7-11 with 3.1 µM M^{pro} on ice (0˚C) are shown. Intermediate species nsp7-8 (yellow), nsp7-9 (cyan), nsp7-11 (steel blue) are colored. The end point at 24 h (incubation at 4˚C) shows mature nsp7 (yellow), nsp8 (green), nsp9 (purple). The following species were only detectable in low intensities, and thus not visible in the shown spectra: nsp8-11, nsp8-9, nsp7-10, nsp10-11, nsp10, Heterotrimer nsp7$_2$+8. Polyprotein processing of HCoV-229E nsp7-11 with authentic termini was performed once with technical triplicates. Source data are provided as a Source Data file.

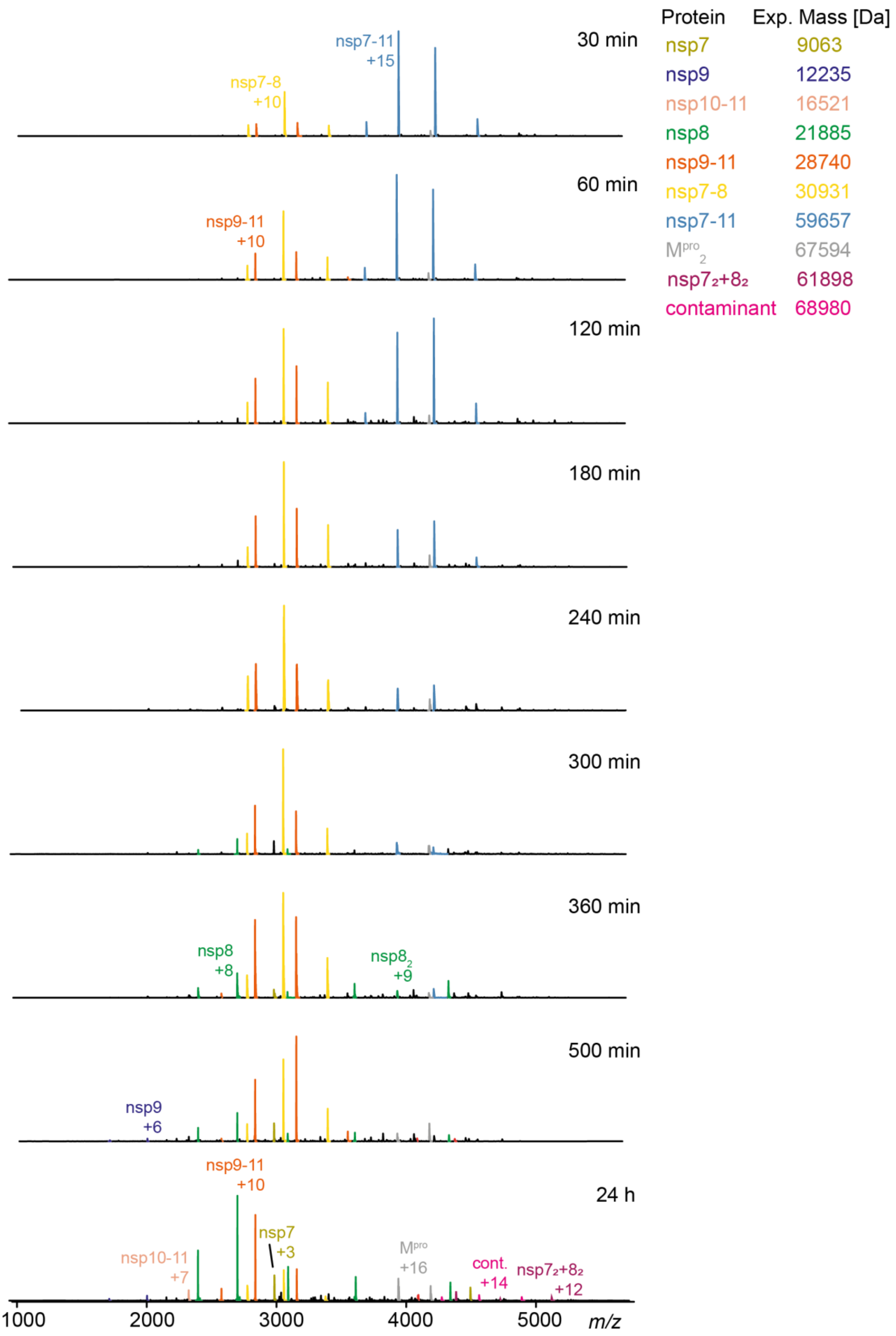

Supplementary Figure S8: Discontinuous processing of MERS-CoV nsp7-11. Representative mass spectra from the time-resolved processing (30-500 min) of 19 µM nsp7-11 with 3.1 µM M^{pro} on ice (0˚C) are shown. Intermediate species nsp9-11 (brick red), nsp7-8 (yellow), nsp7-9 (cyan), nsp7-10 (light blue), nsp7-11 (steel blue) are colored. The end point at 24 h (incubation at 4˚C) shows mature nsp7 (yellow), nsp8 (green), nsp9 (purple), nsp10 (blue) and nsp7$_2$+8$_2$ (purple) heterotetramer. Polyprotein processing of MERS-CoV nsp7-11 with authentic termini was repeated twice with technical triplicates. Source data are provided as a Source Data file.

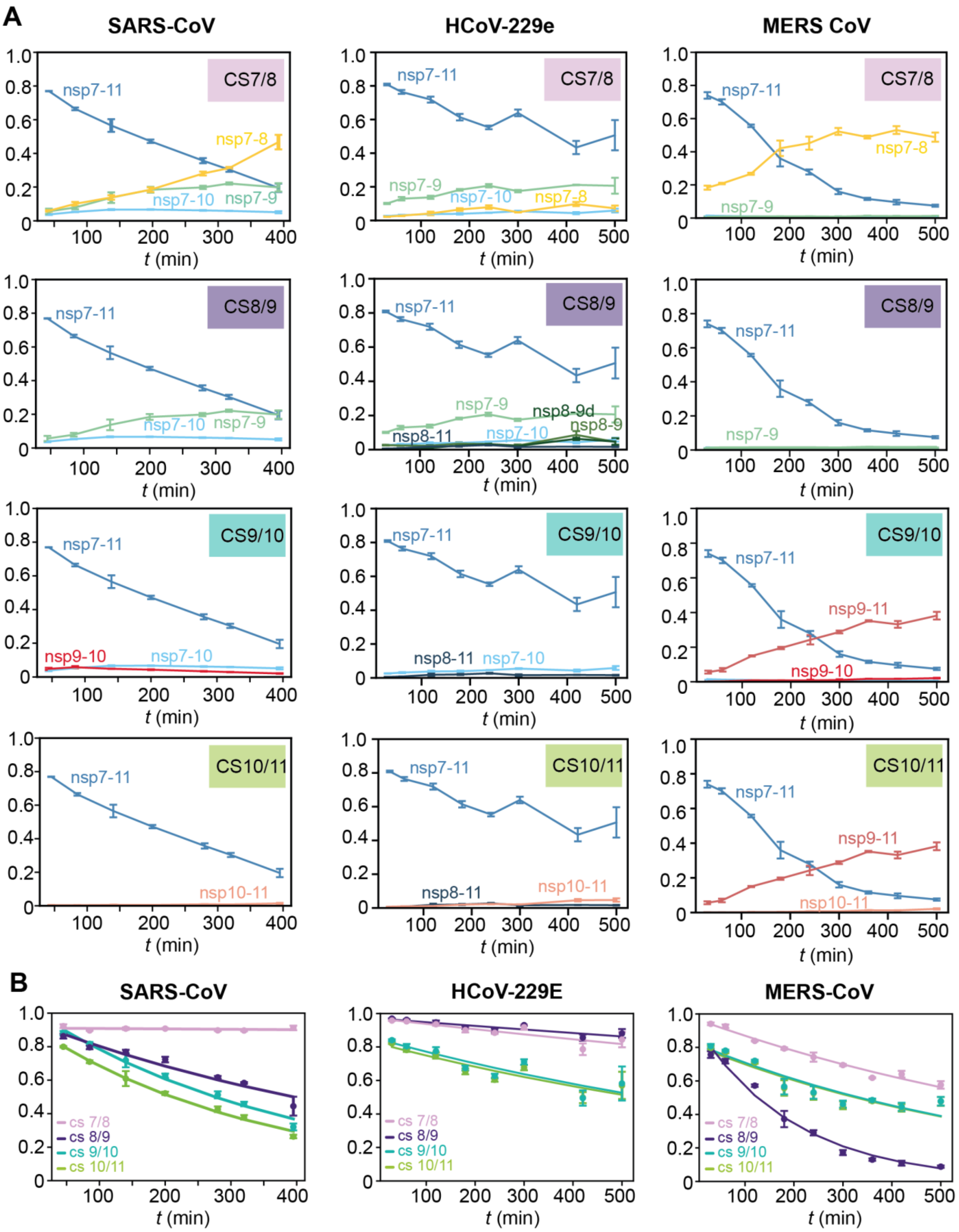

Supplementary Figure S9: Fits for cleavage site rate constants $k_{0^\circ C}$ and corresponding courses of time-resolved intensities per species. Polyprotein processing was performed with nsp7-11 (17 µM-19 µM) and M^{pro} (3.1 µM -3.5 µM) at 0˚C. All intermediate species containing the specific intact cleavage site are summed in order to monitor the conversion of the specific cleavage site. Polyprotein processing was conducted using triplicate measurements, means were calculated and standard errors are depicted as error bars. (**A**) Time-resolved courses of the intermediate species that are considered for each cleavage site are depicted as intensities over time for SARS-CoV, HCoV-229E and MERS-CoV. (**B**) Summed intensities of substrate and intermediates assigned to the corresponding cleavage sites are plotted against time and fitted with first order kinetics. Polyprotein processing of SARS-CoV, SARS-CoV-2 and MERS-CoV nsp7-11 with authentic termini was performed twice with technical triplicates, processing of HCoV-229E nsp7-11 was performed once with technical triplicates. Source data are provided as a Source Data file.

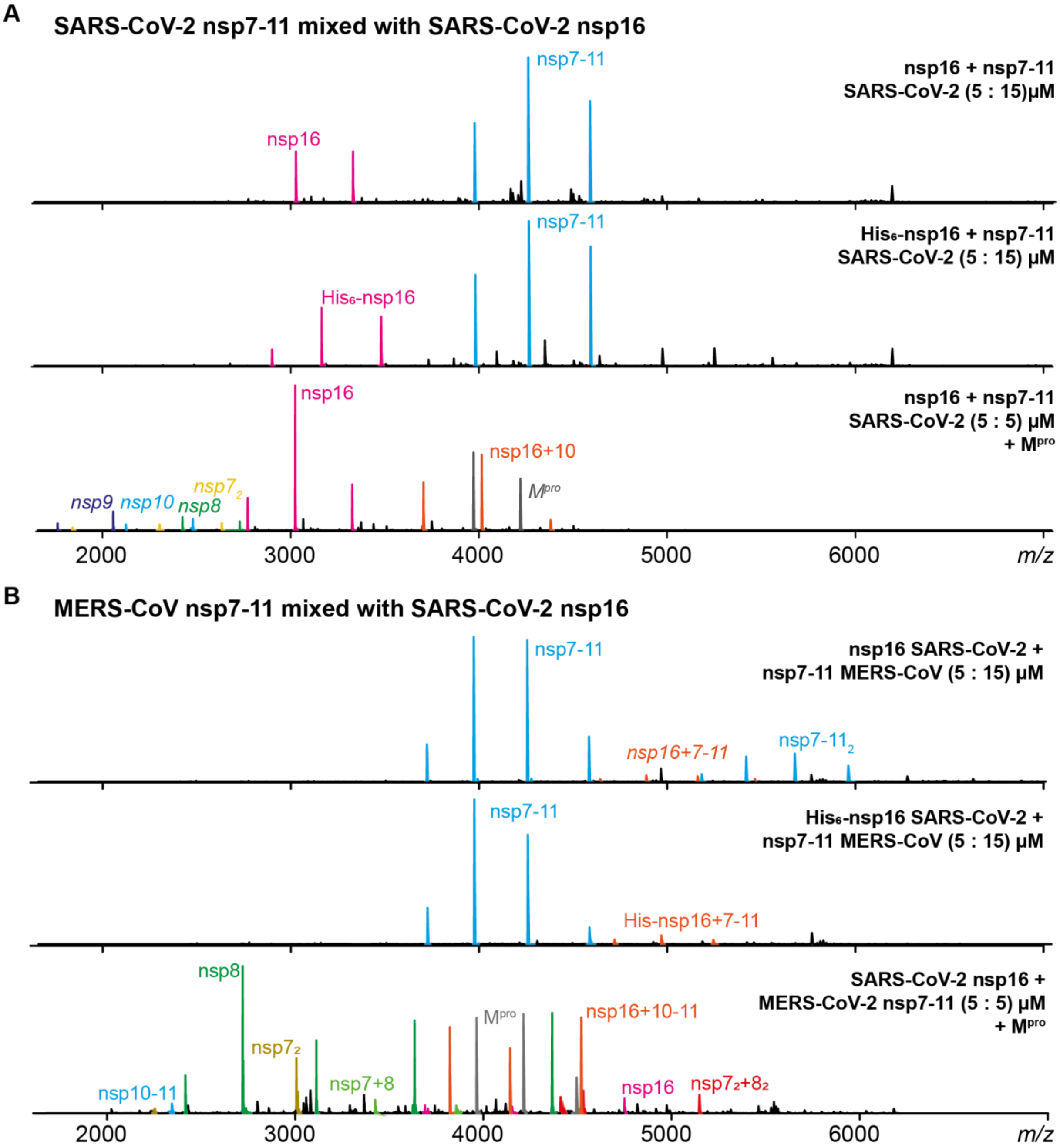

Supplementary Figure S10: Protein-protein interaction of nsp16 with nsp7-11**.** SARS-CoV-2 nsp16 is probed with processed and unprocessed nsp7-11 of SARS-CoV-2 (**A**) and MERS-CoV (**B**). Concentrations are indicated on the right side. His_6-tag of nsp16 contains an M^{pro} cleavage site. In order to rule out any artefacts from the His_6-tag, His_6-nsp16 was incubated with low concentration of M^{pro} to cleave the His_6-tag and obtain nsp16 with authentic termini. Then nsp16 is mixed with the polyprotein. This is shown in the first spectrum in (A) and (B). Complexes of nsp16 are shown in orange. In (A), nsp16 only forms complexes with mature SARS-CoV-2 nsp10 (nsp16+10), whereas in (B) SARS-CoV-2 nsp16 forms complexes with MERS-CoV nsp7-11 as well as nsp10-11. nsp7-11 is colored blue as well as its derived products nsp10 or nsp10-11, nsp16 is shown in pink. Binding experiments for both SARS-CoV and MERS-CoV nsp7-11 were repeated twice with technical triplicates. Source data are provided as a Source Data file.

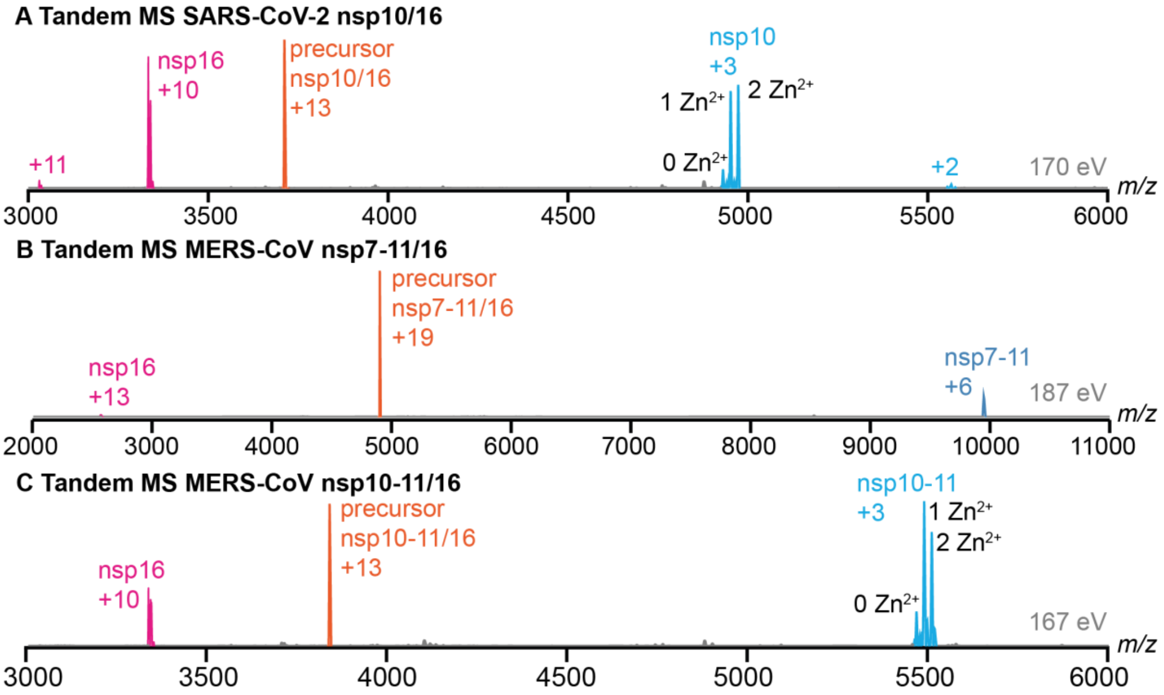

Supplementary Figure S11: Tandem MS of nsp16 complexes. Complex identification was repeated twice. (**A**) Panel shows precursor complex nsp10/16 (orange) of SARS-CoV-2 dissociating in nsp16 (pink) and nsp10 (blue) at 170 eV. (**B**) Precursor peak is the chimeric complex of MERS-CoV nsp7-11/SARS-CoV-2 nsp16. Dissociation of the complex is shown at 187 eV. (**C**) In processed MERS-CoV nsp7-11, nsp10-11 interacts with SARS-CoV-2 nsp16. Precursor peak +13 of nsp10-11/16 (orange) is dissociating at 167 eV. Nsp10 can bind up to two Zn^{2+} giving rise to the observed fine structure as indicated. Some Zn^{2+} remains with nsp16 upon nsp10 dissociation resulting in a double peak. Source data are provided as a Source Data file.

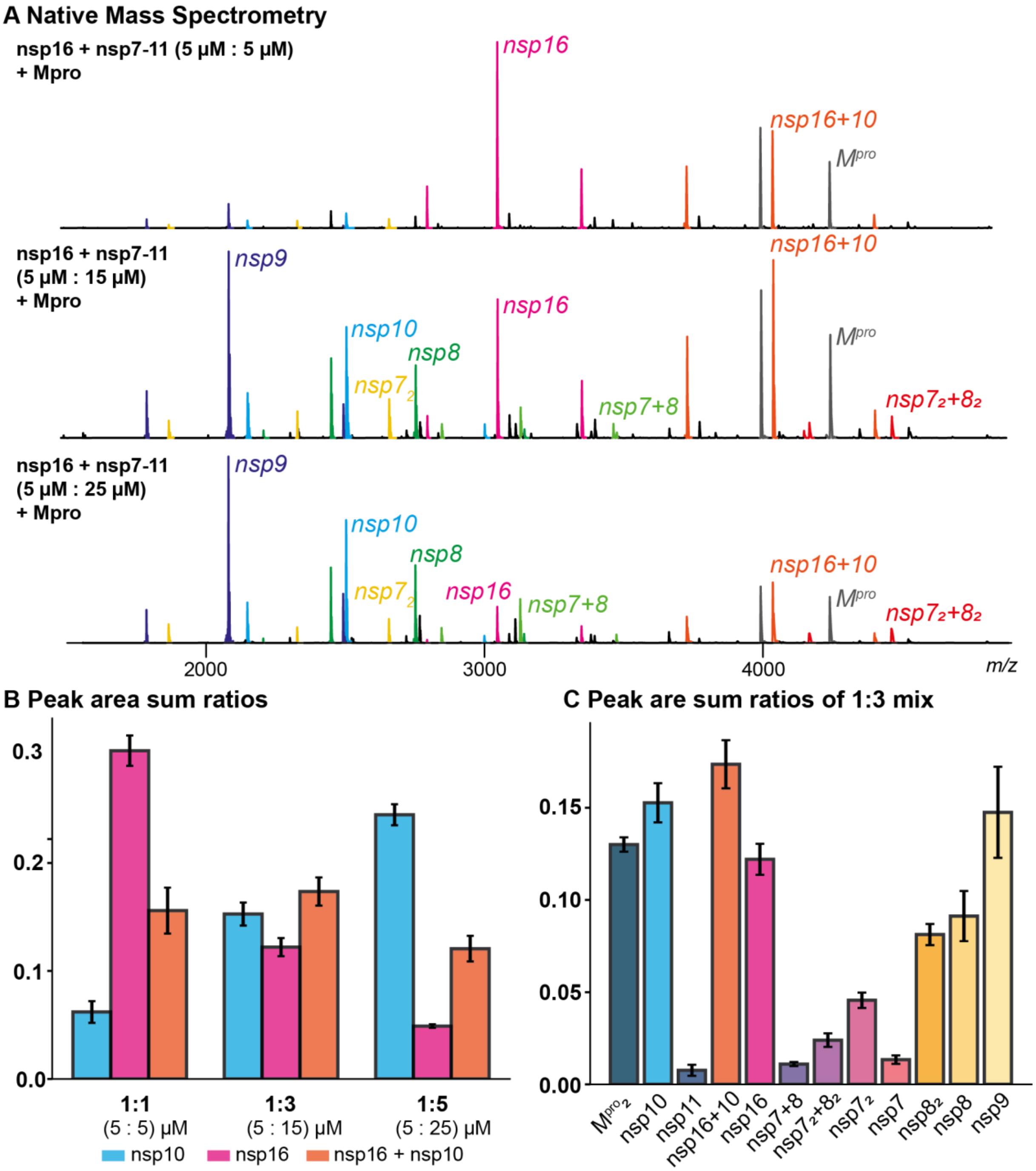

Supplementary Figure S12: Probing affinity of SARS-CoV-2 nsp16+10 complex. Protein complexation of nsp16+10 was investigated at three different concentrations as indicated in the panels. Experiments were repeated twice. Standard error was calculated from triplicate measurements. (A) shows native mass spectra with nsp16+10 in orange, nsp16 in pink and nsp10 in blue. (B) shows the average relative intensity of each species from triplet measurements of the three tested ratios. Peak areas of each corresponding species were considered and normalizd to sum the relative intensities. (C) shows relative intensities of each species in triplcate measurements at a ratio of 5:15 µM. Further nsps are nsp9 (dark blue), nsp7 (yellow), nsp8 (dark green), nsp7+8 heterodimer (light green) and heterotetramer $nsp7_2$+8_2 (red). Source data are provided as a Source Data file.

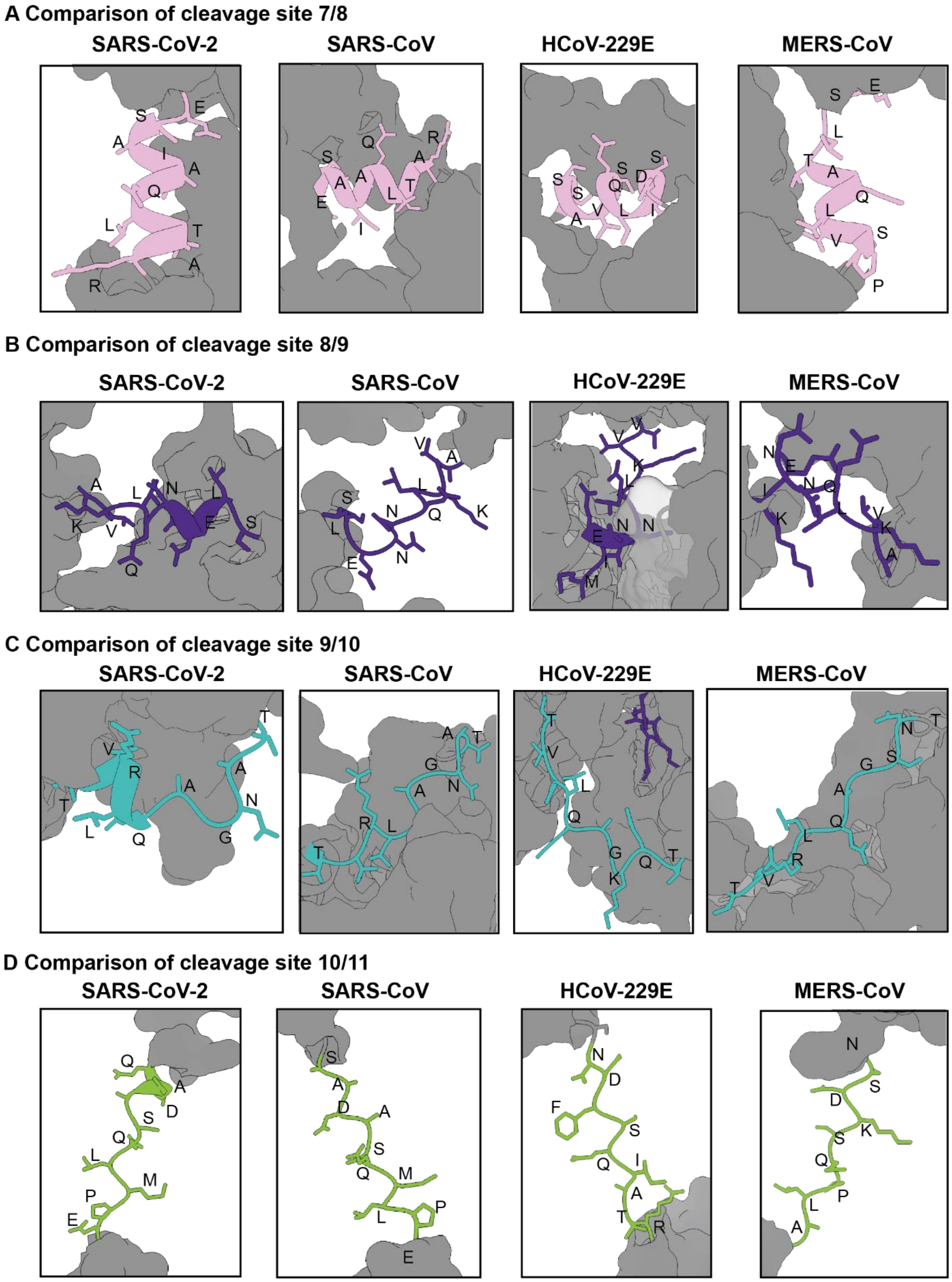

Supplementary Figure S13: Comparison of the four cleavage sites between the four different hCoVs. Zoom in on each predicted cleavage site region P6 to P6'. (A) Predicted α-helical structure spanning CS7/8 (pale pink) is shown in comparison. (B) CS8/9 is shown in purple and predicted to be partly α-helical depending on the CoV species. (C) CS9/10 is depicted in cyan and is predicted to be partly folded as α-helix in SARS-CoV-2. (D) CS10/11 in green is predicted to be disordered and elongated in all four hCoVs. Source data are provided as a Source Data file.

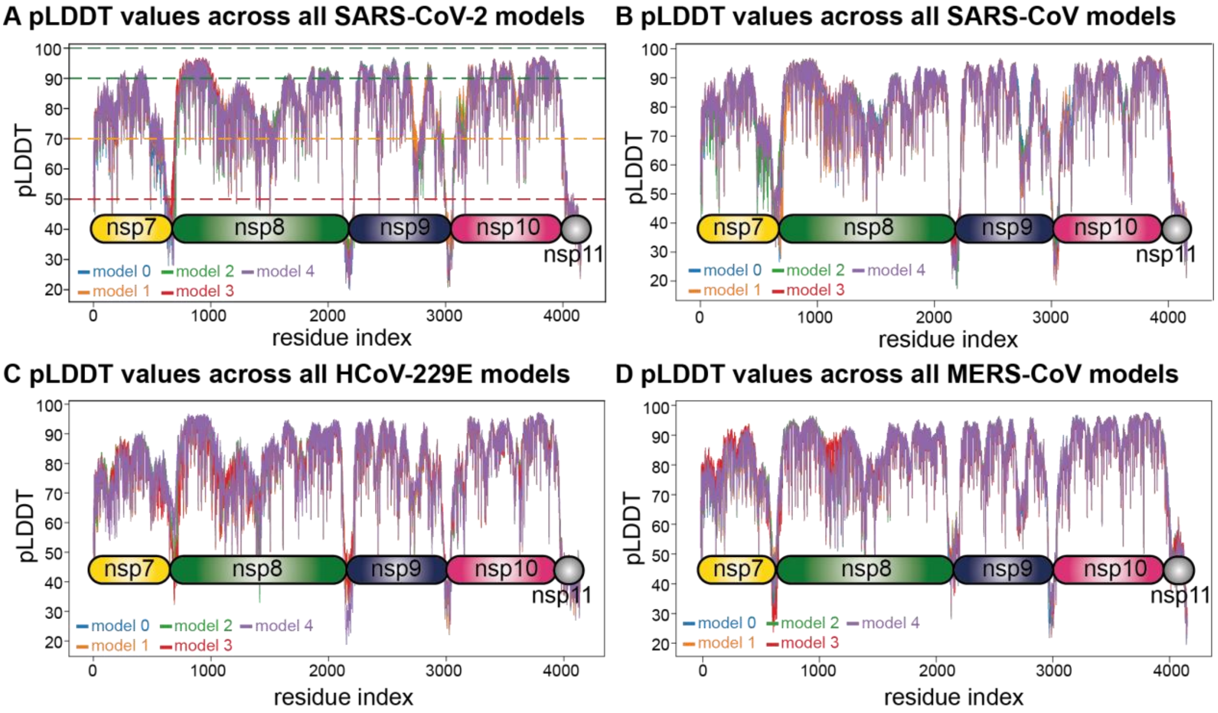

Supplementary Figure S14: Local confidence scores of all AlphaFold 3 models are shown for each CoV: (**A**) SARS-CoV-2, (**B**) SARS-CoV, (**C**) HCoV-229E and (**D**) MERS-CoV. The predicted values of the local distance difference test (pLDDT) are plotted as a function of the residue index. Polyprotein schemes indicate approximate polyprotein region and emphasize low pLDDT values across cleavage sites. Source data are provided as a Source Data file.

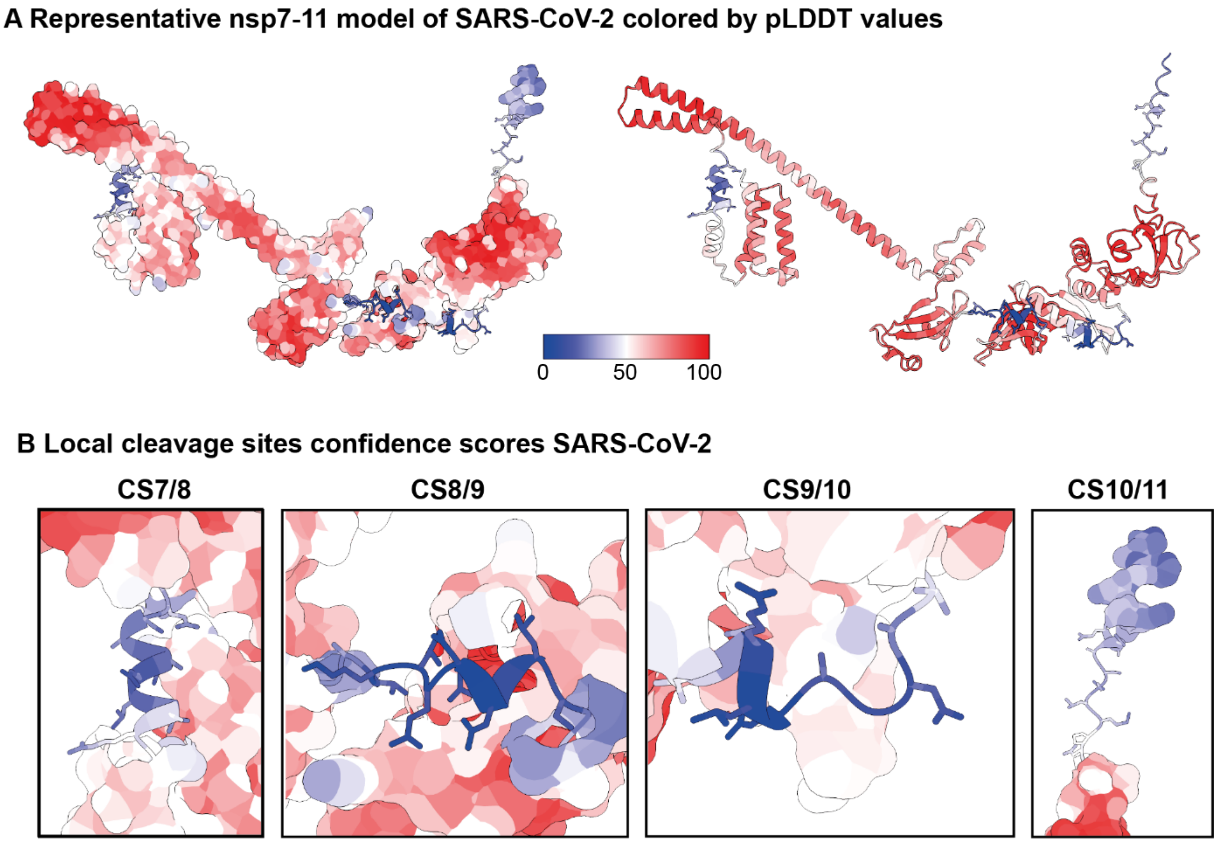

Supplementary Figure S15: Local confidence scores of SARS-CoV-2 model presented on the structure. Predicted values of the local distance difference test (pLDDT) are depicted showing the overall folding (**A**) and the close-ups from the cleavage sites (**B**). The predicted template modeling (pTM) score is 0.33 and has thereby low confidence that the prediction of the folding would resemble the true strucutre. pTM scores above 0.5 are probably close to the real structure. Source data are provided as a Source Data file.

A **Representative nsp7-11 model of SARS-CoV colored by pLDDT values**

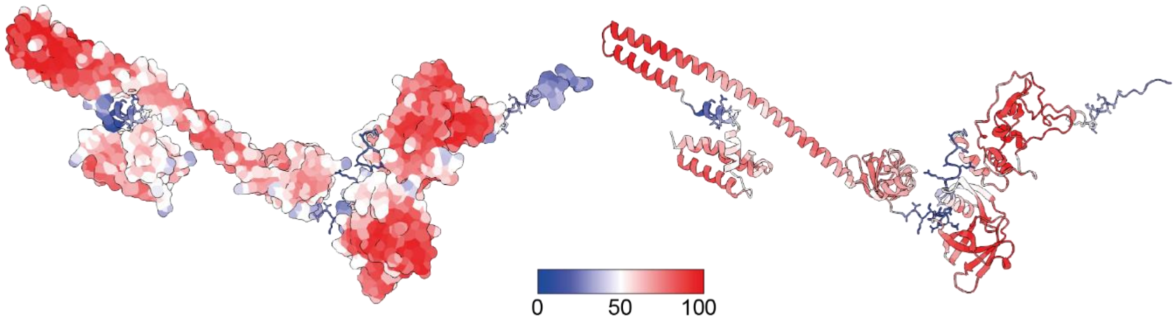

B **Local cleavage sites confidence scores of SARS-CoV**

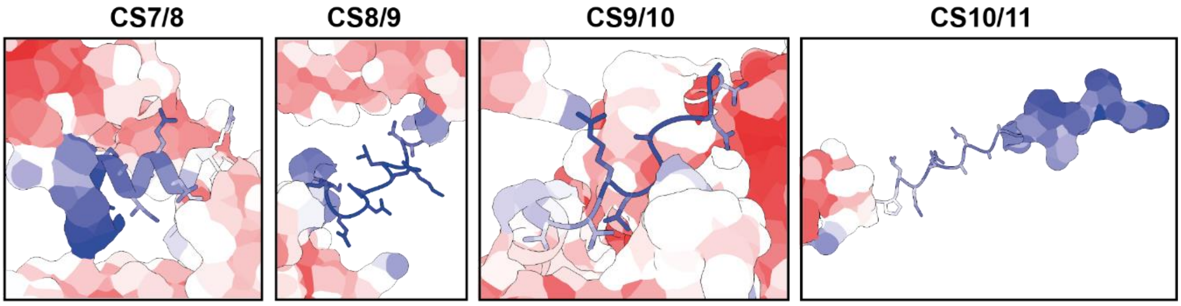

Supplementary Figure S16: Local confidence scores of SARS-CoV model presented on the structure. Overview of model folding is illustrated with and without surface whereby cleavage sites are always shown as ribbon with residue sidechains (**A**). Zoom in on the cleavage site regions are shown in (**B**). pTM-score is low with 0.32 asserting low accuracy of the entire structure. Source data are provided as a Source Data file.

A Representative nsp7-11 model of HCoV-229E colored by pLDDT values

B Local cleavage sites confidence scores HCoV-229E

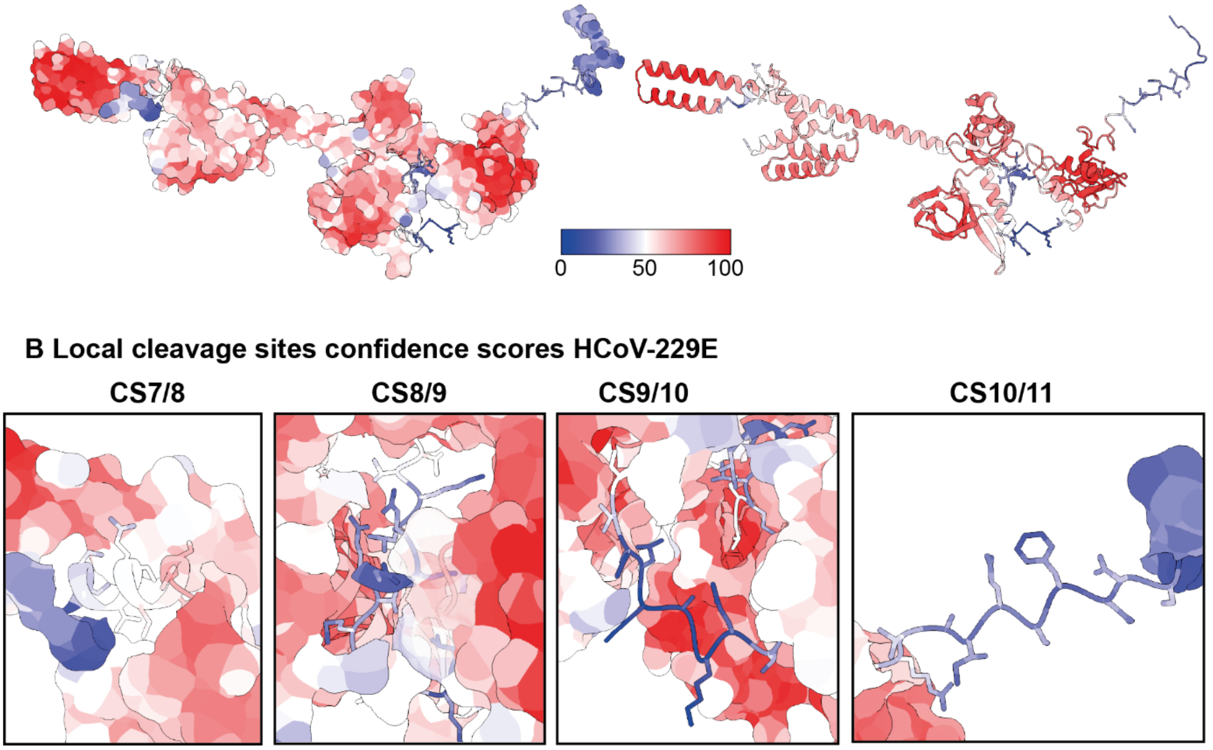

Supplementary Figure S17: Local confidence scores of HCoV-229E model presented on the structure. Overview of model folding is illustrated with and without surface whereby cleavage sites are always shown as ribbon with residue sidechains (**A**). Zoom in on the cleavage site regions are shown in (**B**). pTM-score is low with 0.39 asserting low accuracy of the entire structure. Source data are provided as a Source Data file.

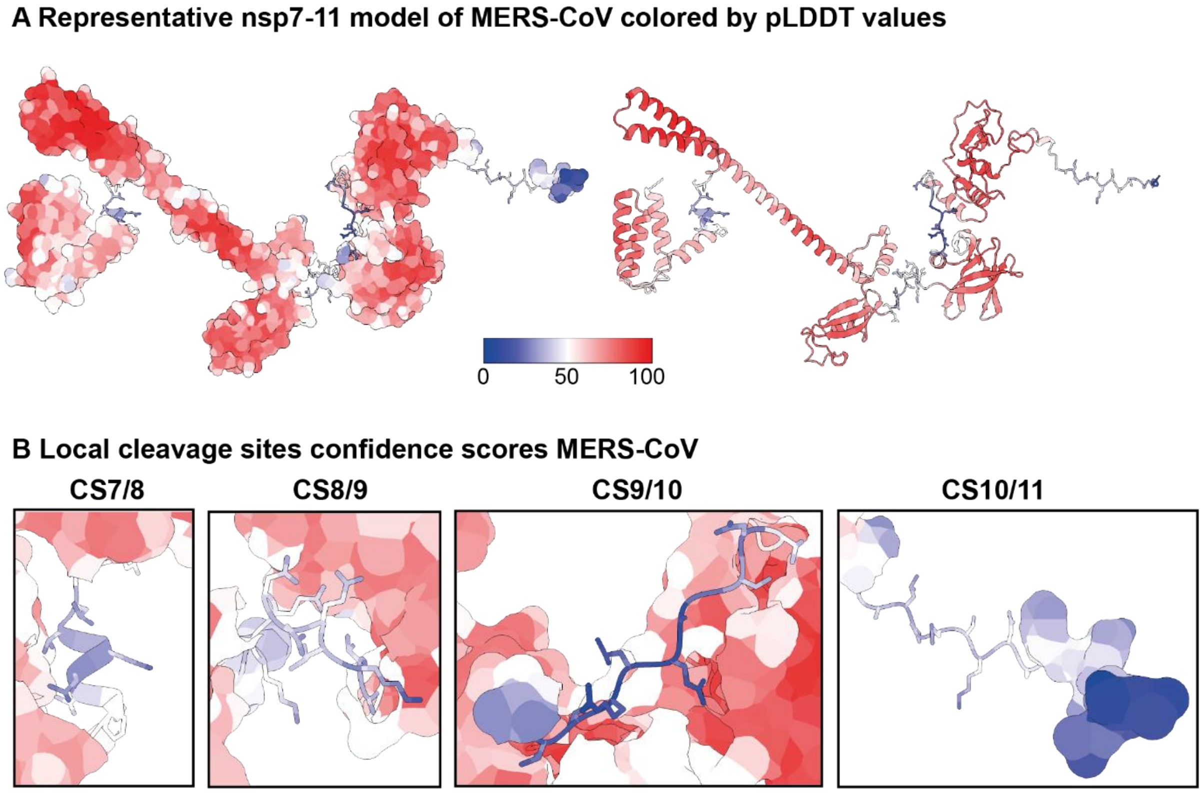

Supplementary Figure S18: Local confidence scores of MERS-CoV model presented on the structure. Overview of model folding is illustrated with and without surface whereby cleavage sites are always shown as ribbon with residue sidechains (**A**). Zoom in on the cleavage site regions are shown in (**B**). pTM-score is low with 0.37 asserting low accuracy of the entire structure. Source data are provided as a Source Data file.

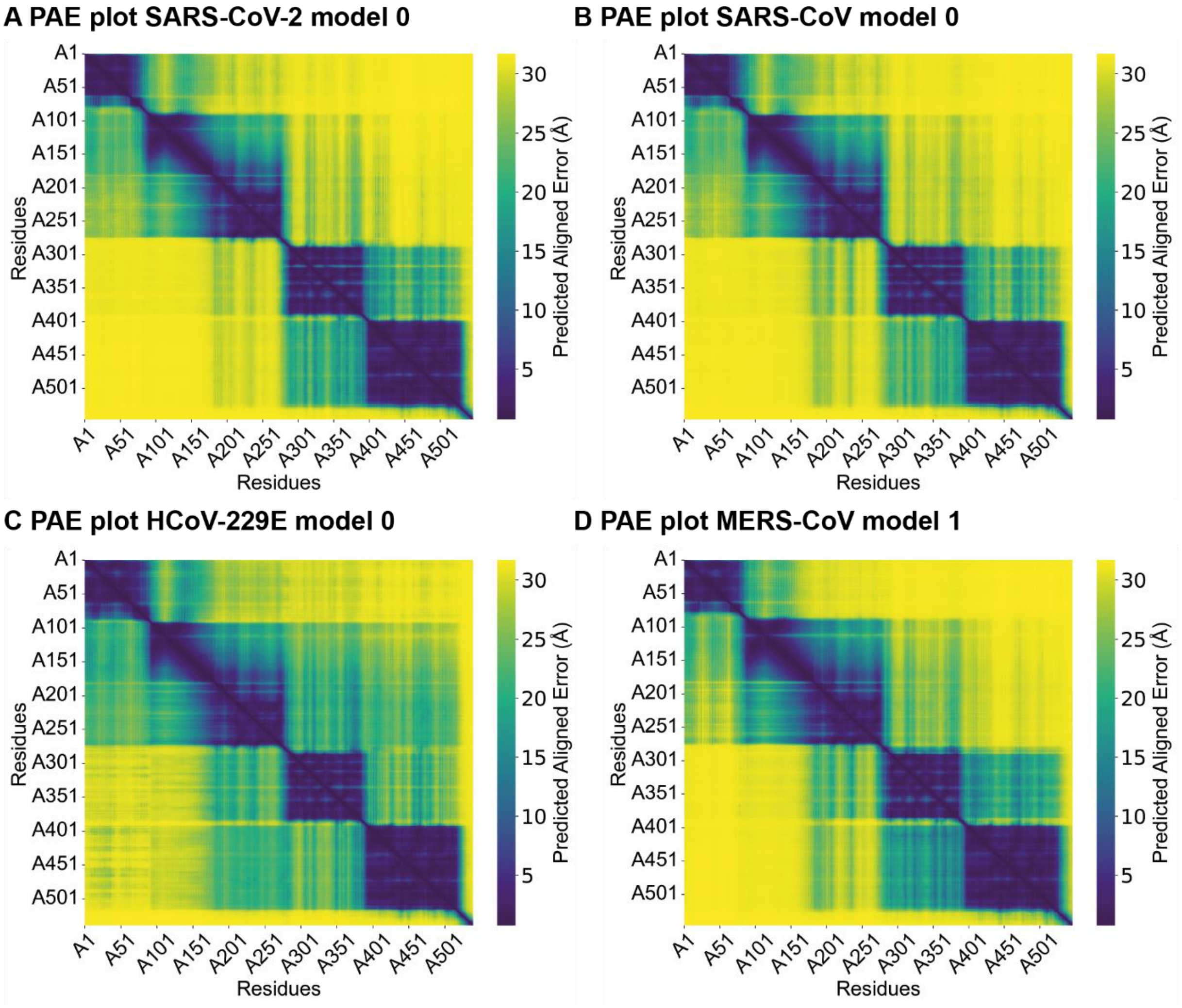

Supplementary Figure S19: Global confidence plots for the selected models. Predicted aligned errors (PAE) in Å are depicted as heatmaps showing higher errors (yellowish-green) around the cleavage site regions and lower errors (blueish) for the indivdual nsp domains. The four panels show PAE plots for SARS-CoV-2 (**A**), SARS-CoV (**B**), HCoV-229E (**C**) and MERS-CoV (**D**). Source data are provided as a Source Data file.

Supplementary Table S1: Measured mass species of nsp7-11C and nsp7-11N processing that were detected on the Q Exactive UHMR Orbitrap. Measured masses were averaged and standard error is given. Source data are provided as a Source Data file.

Name	Theoretical Mass (in Da)	Measured mass (in Da)
nsp7	9,239.8	9,239.0 ± 0.1
nsp7N	10,649.3	10,648.6 ± 1
nsp9	12,378.2	12,378.3 ± 0.2
nsp10+2Zn^{2+}	14,919.9	14,915.7 ± 0.2
nsp10-11+2Zn^{2+}	17,509.7	17,506 ± 1
$nsp7_2$	18,479.6	18,479.8 ± 0.2
nsp8	21,881.1	21,880.4 ± 0.1
nsp9-10+2Zn^{2+}	27,280.1	27,276.6 ± 0.1
nsp9-11+2Zn^{2+}	29,869.8	29,866.2 ± 0.2
nsp7-8	31,102.9	31,103 ± 1
nsp7+8	31,120.9	31,120 ± 1
nsp7-8N	32,512.3	32,512 ± 1
nsp7N+8	32,530.4	32,530 ± 1.2
M^{pro}	33,668.5	33,796 ± 1
nsp7-9	43,463.1	43,462 ± 1
$nsp8_2$	43,762.2	43,762 ± 2
nsp7-10C+2Zn^{2+}	58,365.0	58,360 ± 2
nsp7-10N+2Zn^{2+}	59,774.7	59,774 ± 3
nsp7-11C+2Zn^{2+}	60,954.7	60,950 ± 4
nsp7-11N+2Zn^{2+}	61,082.9	61,085 ± 1
$nsp7_2+8_2$	62,241.8	62,244 ± 5
nsp7N+7C +8_2	63,651.3	63,655 ± 3
$nsp7N_2+8_2$	65,024.6	65,061 ± 3
M^{pro}_2	67,337.0	67,598 ± 1

Supplementary Table S2: Measured masses of SARS-CoV-2 nsps and their complexes determined by native MS. Masses were determined from representative mass spectra. Uncertainty was computed as standard error. Masses are reported in Daltons (Da). Source data are provided as a Source Data file.

Virus strain	Protein	Theoretical Mass (Da)	Measured Mass (Da)
SARS-CoV-2	nsp11	1325.6	1325.654 ± 0.001
SARS-CoV-2	nsp11 + Na^+	1349.4	1347.63 ± 0.01
SARS-CoV-2	nsp7	9239.8	9239.1 ± 0.2
SARS-CoV-2	nsp9	12378.2	12377.6 ± 0.2
SARS-CoV-2	nsp10+2Zn^{2+}	14789.9	14916.3 ± 0.2
SARS-CoV-2	nsp10-11+2Zn^{2+}	16228.3	16224 ± 1
SARS-CoV-2	nsp8	21881.1	21880.7 ± 0.2
SARS-CoV-2	nsp9-10+2Zn^{2+}	27280.1	27275.9 ± 0.2
SARS-CoV-2	nsp9-11+2Zn^{2+}	28588.5	28584.7 ± 0.5
SARS-CoV-2	nsp7-8	31102.9	31102.3 ± 0.3
SARS-CoV-2	nsp7+8	31120.9	31102.7 ± 0.3
SARS-CoV-2	nsp7-9	43463.1	43460 ± 2
SARS-CoV-2	$nsp8_2$	43762.2	43761 ± 2
SARS-CoV-2	nsp7-10+2Zn^{2+}	58235	58366 ± 1
SARS-CoV-2	nsp7-11+2Zn^{2+}	59543.4	59677 ± 3
SARS-CoV-2	$nsp7_2$+8_2	62241.6	62244 ± 2

Supplementary Table S3: Measured masses of SARS-CoV nsps and their complexes determined by native MS. Masses were determined from representative mass spectra. Uncertainty was computed as standard error. Masses are reported in Daltons (Da). Source data are provided as a Source Data file.

Virus strain	Protein	Theoretical Mass (Da)	Measured Mass (Da)
SARS-CoV	nsp7	9267.8	9267.3 ± 0.2
SARS-CoV	nsp9	12401.2	12400.8 ± 0.3
SARS-CoV	nsp10+2Zn^{2+}	14844	14970.4 ± 0.3
SARS-CoV	nsp10-11+2Zn^{2+}	16125.4	16233.1 ± 0.5
SARS-CoV	$nsp7_2$	18535.7	18535 ± 1
SARS-CoV	nsp8	21866	21865.7 ± 0.3
SARS-CoV	nsp9-10+2Zn^{2+}	27227.1	27353 ± 3
SARS-CoV	nsp9-11+2Zn^{2+}	28508.5	28635.3 ± 0.8
SARS-CoV	nsp7-8	31115.9	31115.7 ± 0.2
SARS-CoV	nsp7-9	43499	43497 ± 2
SARS-CoV	$nsp8_2$	43732.1	43731 ± 1
SARS-CoV	nsp7-10+2Zn^{2+}	58325	58454 ± 2
SARS-CoV	nsp7-11+2Zn^{2+}	59606.3	59753 ± 4
SARS-CoV	$nsp7_2$+8_2	62267.7	62264 ± 4

Supplementary Table S4: Measured masses of MERS-CoV nsps and their complexes determined by native MS. Masses were determined from representative mass spectra. Uncertainty was computed as standard error. Masses are reported in Daltons (Da). Source data are provided as a Source Data file.

Virus strain	Protein	Theoretical Mass (Da)	Measured Mass (Da)
MERS-CoV	nsp11	1522.7	1522.80 ± 0.01
MERS-CoV	nsp11+Na^+	1545.7	1543.79 ± 0.01
MERS-CoV	nsp7	9063.4	9063.2 ± 0.7
MERS-CoV	nsp9	12236.2	12235.2 ± 0.4
MERS-CoV	nsp10+2Zn^{2+}	15020.9	15018 ± 2
MERS-CoV	nsp10-11+2Zn^{2+}	16525.6	16521.8 ± 0.3
MERS-CoV	nsp8	21886.2	21885.4 ± 0.2
MERS-CoV	nsp9-11+2Zn^{2+}	28743.8	28741 ± 1
MERS-CoV	nsp7-8	30931.5	30931.1 ± 0.2
MERS-CoV	nsp$7_2$8	40012.9	40011 ± 1
MERS-CoV	nsp7-9	43149.7	43149 ± 1
MERS-CoV	nsp8_2	43772.4	43770.2 ± 0.5
MERS-CoV	nsp7-11+2Zn^{2+}	59527.3	59654 ± 1
MERS-CoV	nsp$7_2 8_2$	61899.4	61898 ± 4

Supplementary Table S5: Measured masses of SARS-CoV-2 nsps and their complexes determined by native MS. Masses were determined from representative mass spectra. Uncertainty was computed as standard error. Masses are reported in Daltons (Da). Source data are provided as a Source Data file.

Virus strain	Protein	Theoretical Mass (Da)	Measured Mass
HCoV-229E	nsp11	1841.0	1841.92 ± 0.03
HCoV-229E	nsp7	9300.6	9299.8 ± 0.8
HCoV-229E	nsp9	12045.9	12045.5 ± 0.2
HCoV-229E	nsp10+2Zn^{2+}	14525.4	14521 ± 1
HCoV-229E	nsp10-11+2Zn^{2+}	16348.3	16345 ± 6
HCoV-229E	nsp7_2	18601.3	18600.5 ± 0.3
HCoV-229E	nsp8	21623.1	21622.9 ± 0.3
HCoV-229E	nsp8_2	24091.7	43248 ± 1
HCoV-229E	nsp7-8	30905.7	30908 ± 3
HCoV-229E	nsp8-9	33651	33651 ± 1
HCoV-229E	nsp$7_2$8	40224.4	40224 ± 4
HCoV-229E	nsp7-9	42933.6	42932 ± 2
HCoV-229E	nsp8-11+2Zn^{2+}	49981.3	49992 ± 16
HCoV-229E	nsp7-10+2Zn^{2+}	57440.9	57440.9 ± 0.4
HCoV-229E	nsp7-11+2Zn^{2+}	59263.9	59256 ± 25
HCoV-229E	nsp8-9_2	67302	67296 ±10

Supplementary Table S6: Measured mass of SARS-CoV-2 and MERS-CoV nsp7-11 and their complexes with SARS-CoV-2 nsp16 determined by native MS**.** Masses were determined from representative mass spectra. Uncertainty was computed as standard error. Masses are reported in Daltons (Da). Source data are provided as a Source Data file.

Virus strain	Protein	Theoretical Mass (Da)	Measured Mass ± Uncertainty (Da)
SARS-CoV-2	nsp7	9239.8	9239.25 ± 0.23
SARS-CoV-2	nsp9	12378.2	12377.55 ± 0.12
SARS-CoV-2	nsp10+Zn^{2+}	14920.7	14918 ± 4
SARS-CoV-2	$nsp7_2$	18479.6	18478 ± 1
SARS-CoV-2	nsp8	21881.1	21881.04 ± 0.12
SARS-CoV-2	$nsp9_2$	24756.4	24755.3 ± 0.6
SARS-CoV-2	nsp7-8/nsp7+8	31120.9	31111 ± 10
SARS-CoV-2	nsp16	33323.3	33323.27 ± 0.14
SARS-CoV-2	nsp16-His_6	34644.8	34774 ± 1
SARS-CoV-2	nsp16+10+2Zn^{2+}	48224.0	48236 ± 1
SARS-CoV-2	nsp7-11+2Zn^{2+}	59674.2	59674 ± 3
SARS-CoV-2	$nsp7_2$+8_2	62241.6	62251 ± 8
SARS-CoV-2	nsp7-11+2Zn^{2+}+16	92997.5	93008 ± 6
MERS-CoV	nsp9	12236.2	12235.93 ± 0.24
MERS-CoV	nsp10-11+2Zn^{2+}	16526.4	16522 ± 1
MERS-CoV	$nsp7_2$	18126.8	18126.01 ± 0.34
MERS-CoV	nsp8	21886.2	21886.22 ± 0.13
MERS-CoV	nsp7+8	30949.6	30949.87 ± 0.43
MERS-CoV	nsp16	33323.3	33322 ± 1
MERS-CoV	nsp16-His_6	34644.8	34775 ± 1
MERS-CoV	$nsp7_2$8	40012.9	40014 ± 1
MERS-CoV	nsp16+10-11+2Zn^{2+}	49849.7	49848 ± 2
MERS-CoV	nsp7-11+2Zn^{2+}	59658.0	59658 ± 4
MERS-CoV	$nsp7_2 8_2$	61899.4	61898 ± 7
MERS-CoV	nsp7	9063.4	9062.88 ± 0.20
MERS-CoV	nsp7-11+2Zn^{2+}+16	92981.4	93001 ± 22
MERS-CoV	nsp7-11+2Zn^{2+}+16-His_6	94302.8	94449 ± 7

Supplementary Table S7: Amino acid sequences of all recombinantly expressed proteins and their theoretical mass (in Da). The constructs that have a cleavable tag are shown with their authentic sequences. All of them are expressed with an N-terminal Sumo-Strep2-His_6-tag that is listed once for all.

Protein	Sequence	Theoretical Mass (in Da)
**nsp7-11C**	SKMSDVKCTSVVLLSVLQQLRVESSSKLWAQCVQLHND ILLAKDTTEAFEKMVSLLSVLLSMQGAVDINKLCEEMLDN RATLQAIASEFSSLPSYAAFATAQEAYEQAVANGDSEVV LKKLKKSLNVAKSEFDRDAAMQRKLEKMADQAMTQMY KQARSEDKRAKVTSAMQTMLFTMLRKLDNDALNNIINNA RDGCVPLNIIPLTTAAKLMVVIPDYNTYKNTCDGTTFTYA SALWEIQQVVDADSKIVQLSEISMDNSPNLAWPLIVTALR ANSAVKLQNNELSPVALRQMSCAAGTTQTACTDDNALA YYNTTKGGRFVLALLSDLQDLKWARFPKSDGTGTIYTEL EPPCRFVTDTPKGPKVKYLYFIKGLNNLNRGMVLGSLAA TVRLQAGNATEVPANSTVLSFCAFAVDAAKAYKDYLASG GQPITNCVKMLCTHTGTGQAITVTPEANMDQESFGGAS CCLYCRCHIDHPNPKGFCDLKGKYVQIPTTCANDPVGFT LKNTVCTVCGMWKGYGCSCDQLREPMLQSADAQSFLN GFAVSARGSHHHHHH	60,824
**nsp7-11N**	ASRGSHHHHHHGASKMSDVKCTSVVLLSVLQQLRVESS SKLWAQCVQLHNDILLAKDTTEAFEKMVSLLSVLLSMQG AVDINKLCEEMLDNRATLQAIASEFSSLPSYAAFATAQEA YEQAVANGDSEVVLKKLKKSLNVAKSEFDRDAAMQRKL EKMADQAMTQMYKQARSEDKRAKVTSAMQTMLFTMLR KLDNDALNNIINNARDGCVPLNIIPLTTAAKLMVVIPDYNT YKNTCDGTTFTYASALWEIQQVVDADSKIVQLSEISMDN SPNLAWPLIVTALRANSAVKLQNNELSPVALRQMSCAAG TTQTACTDDNALAYYNTTKGGRFVLALLSDLQDLKWAR FPKSDGTGTIYTELEPPCRFVTDTPKGPKVKYLYFIKGLN NLNRGMVLGSLAATVRLQAGNATEVPANSTVLSFCAFA VDAAKAYKDYLASGGQPITNCVKMLCTHTGTGQAITVTP EANMDQESFGGASCCLYCRCHIDHPNPKGFCDLKGKYV QIPTTCANDPVGFTLKNTVCTVCGMWKGYGCSCDQLRE PMLQSADAQSFLNGFAV	60,953
**Sumo-Strep2-His6-tag**	MGSSHHHHHHSSGWSHPQFEKGGMSDSEVNQEAKPE VKPEVKPETHINLKVSDGSSEIFFKIKKTTPLRRLMEAFAK RQGKEMDSLRFLYDGIRIQADQTPEDLDMEDNDIIEAHR EQIGG	13,832
**nsp7-11 SARS-CoV-2**	SKMSDVKCTSVVLLSVLQQLRVESSSKLWAQCVQLHND ILLAKDTTEAFE KMVSLLSVLLSMQGAVDINKLCEEMLDNRATLQAIASEF SSLPSYAAFATAQEAYEQAVANGDSEVVLKKLKKSLNVA KSEFDRDAAMQRKLEKMADQAMTQMYKQARSEDKRAK VTSAMQTMLFTMLRKLDNDALNNIINNARDGCVPLNIIPL TTAAKLMVVIPDYNTYKNTCDGTTFTYASALWEIQQVVD ADSKIVQLSEISMDNSPNLAWPLIVTALRANSAVKLQNNE LSPVALRQMSCAAGTTQTACTDDNALAYYNTTKGGRFV LALLSDLQDLKWARFPKSDGTGTIYTELEPPCRFVTDTP KGPKVKYLYFIKGLNNLNRGMVLGSLAATVRLQAGNATE VPANSTVLSFCAFAVDAAKAYKDYLASGGQPITNCVKML CTHTGTGQAITVTPEANMDQESFGGASCCLYCRCHIDH PNPKGFCDLKGKYVQIPTTCANDPVGFTLKNTVCTVCG MWKGYGCSCDQLREPMLQSADAQSFLNGFAV	59,543

**nsp7-11 SARS-CoV**	SKMSDVKCTSVVLLSVLQQLRVESSSKLWAQCVQLHND ILLAKDTTEAFEKMVSLLSVLLSMQGAVDINRLCEEMLDN RATLQAIASEFSSLPSYAAYATAQEAYEQAVANGDSEVV LKKLKKSLNVAKSEFDRDAAMQRKLEKMADQAMTQMY KQARSEDKRAKVTSAMQTMLFTMLRKLDNDALNNIINNA RDGCVPLNIIPLTTAAKLMVVVPDYGTYKNTCDGNTFTY ASALWEIQQVVDADSKIVQLSEINMDNSPNLAWPLIVTAL RANSAVKLQNNELSPVALRQMSCAAGTTQTACTDDNAL AYYNNSKGGRFVLALLSDHQDLKWARFPKSDGTGTIYT ELEPPCRFVTDTPKGPKVKYLYFIKGLNNLNRGMVLGSL AATVRLQAGNATEVPANSTVLSFCAFAVDPAKAYKDYLA SGGQPITNCVKMLCTHTGTGQAITVTPEANMDQESFGG ASCCLYCRCHIDHPNPKGFCDLKGKYVQIPTTCANDPVG FTLRNTVCTVCGMWKGYGCSCDQLREPLMQSADASTF LNGFAV	59,606
**nsp7-11 MERS-CoV**	SKLTDLKCTSVVLLSVLQQLHLEANSRAWAFCVKCHNDI LAATDPSEAFEKFVSLFATLMTFSGNVDLDALASDIFDTP SVLQATLSEFSHLATFAELEAAQKAYQEAMDSGDTSPQ VLKALQKAVNIAKNAYEKDKAVARKLERMADQAMTSMY KQARAEDKKAKIVSAMQTMLFGMIKKLDNDVLNGIISNAR NGCIPLSVIPLCASNKLRVVIPDFTVWNQVVTYPSLNYAG ALWDITVINNVDNEIVKSSDVVDSNENLTWPLVLECTRAS TSAVKLQNNEIKPSGLKTMVVSAGQEQTNCNTSSLAYYE PVQGRKMLMALLSDNAYLKWARVEGKDGFVSVELQPP CKFLIAGPKGPEIRYLYFVKNLNNLHRGQVLGHIAATVRL QAGSNTEFASNSSVLSLVNFTVDPQKAYLDFVNAGGAP LTNCVKMLTPKTGTGIAISVKPESTADQETYGGASVCLY CRAHIEHPDVSGVCKYKGKFVQIPAQCVRDPVGFCLSN TPCNVCQYWIGYGCNCDSLRQAALPQSKDSNFLNESGV LL	59,527
**nsp7-11 HCoV-229E**	SKLTDLKCTNVVLMGILSNMNIASNSKEWAYCVEMHNKI NLCDDPETAQELLLALLAFFLSKHSDFGLGDLVDSYFEN DSILQSVASSFVGMPSFVAYETARQEYENAVANGSSPQI IKQLKKAMNVAKAEFDRESSVQKKINRMAEQAAAAMYK EARAVNRKSKVVSAMHSLLFGMLRRLDMSSVDTILNMA RNGVVPLSVIPATSAARLVVVVPDHDSFVKMMVDGFVH YAGVVWTLQEVKDNDGKNVHLKDVTKENQEILVWPLILT CERVVKLQNNEIMPGKMKVKATKGEGDGGITSEGNALY NNEGGRAFMYAYVTTKPGMKYVKWEHDSGVVTVELEP PCRFVIDTPTGPQIKYLYFVKNLNNLRRGAVLGYIGATVR LQAGKQTEFVSNSHLLTHCSFAVDPAAAYLDAVKQGAK PVGNCVKMLTNGSGSGQAITCTIDSNTTQDTYGGASVCI YCRAHVAHPTMDGFCQYKGKWVQVPIGTNDPIRFCLEN TVCKVCGCWLNHGCTCDRTAIQSFDNSYLNESGALVPL D	59,134
**nsp16**	MHHHHHHSAVLQSSQAWQPGVAMPNLYKMQRMLLEK CDLQNYGDSATLPKGIMMNVAKYTQLCQYLNTLTLAVPY NMRVIHFGAGSDKGVAPGTAVLRQWLPTGTLLVDSDLN DFVSDADSTLIGDCATVHTANKWDLIISDMYDPKTKNVT KENDSKEGFFTYICGFIQQKLALGGSVAIKITEHSWNADL YKLMGHFAWWTAFVTNVNASSSEAFLIGCNYLGKPREQ IDGYVMHANYIFWRNTNPIQLSSYSLFDMSKFPLKLRGT AVMSLKEGQINDMILSLLSKGRLIIRENNRVVISSDVLVNN	34,776
**M^{pro}**	SGFRKMAFPSGKVEGCMVQVTCGTTTLNGLWLDDVVY CPRHVICTSEDMLNPNYEDLLIRKSNHNFLVQAGNVQLR VIGHSMQNCVLKLKVDTANPKTPKYKFVRIQPGQTFSVL	33,669

	ACYNGSPSGVYQCAMRPNFTIKGSFLNGSCGSVGFNID YDCVSFCYMHHMELPTGVHAGTDLEGNFYGPFVDRQT AQAAGTDTTITVNVLAWLYAAVINGDRWFLNRFTTTLND FNLVAMKYNYEPLTQDHVDILGPLSAQTGIAVLDMCASL KELLQNGMNGRTILGSALLEDEFTPFDVVRQCSGVTFQ	

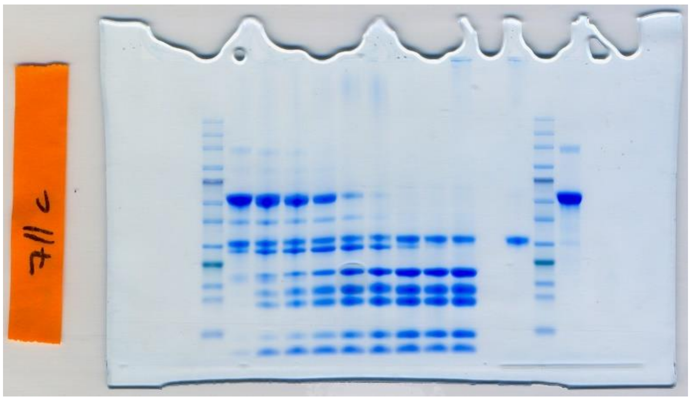

Supplementary Figure S20: Raw image of SDS-PAGE from nsp7-11C processing

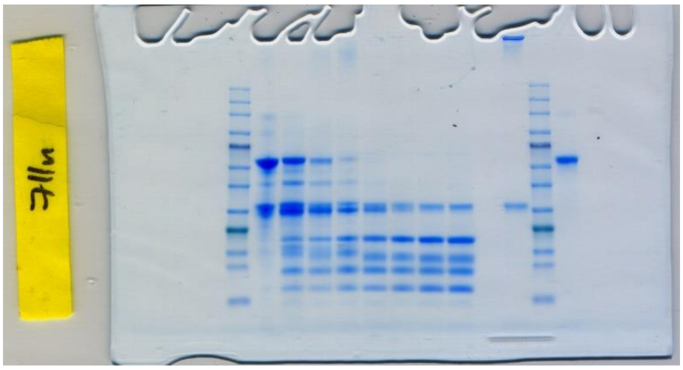

Supplementary Figure S21: Raw image of SDS-PAGE from nsp7-11N processing
