# Supplementary material for: The kinetics of nsp7-11 polyprotein processing and impact on complexation with nsp16 among human coronaviruses
Source: Nat Commun. 2025 Sep 9;16:8244. doi: 10.1038/s41467-025-61554-y (PMC12420816; doi:10.1038/s41467-025-61554-y)
Supplement: Supplementary file 2 — Reporting summary [file 41467_2025_61554_MOESM2_ESM.pdf]

nature portfolio

Corresponding author(s): Charlotte Uetrecht

Last updated by author(s): Jun 19, 2025

Reporting Summary

Nature Portfolio wishes to improve the reproducibility of the work that we publish. This form provides structure for consistency and transparency in reporting. For further information on Nature Portfolio policies, see our Editorial Policies and the Editorial Policy Checklist.

Statistics

For all statistical analyses, confirm that the following items are present in the figure legend, table legend, main text, or Methods section.

n/a	Confirmed	
☐	☒	The exact sample size (*n*) for each experimental group/condition, given as a discrete number and unit of measurement
☐	☒	A statement on whether measurements were taken from distinct samples or whether the same sample was measured repeatedly
☒	☐	The statistical test(s) used AND whether they are one- or two-sided<br>*Only common tests should be described solely by name; describe more complex techniques in the Methods section.*
☒	☐	A description of all covariates tested
☐	☒	A description of any assumptions or corrections, such as tests of normality and adjustment for multiple comparisons
☐	☒	A full description of the statistical parameters including central tendency (e.g. means) or other basic estimates (e.g. regression coefficient) AND variation (e.g. standard deviation) or associated estimates of uncertainty (e.g. confidence intervals)
☒	☐	For null hypothesis testing, the test statistic (e.g. *F*, *t*, *r*) with confidence intervals, effect sizes, degrees of freedom and *P* value noted<br>*Give P values as exact values whenever suitable.*
☒	☐	For Bayesian analysis, information on the choice of priors and Markov chain Monte Carlo settings
☒	☐	For hierarchical and complex designs, identification of the appropriate level for tests and full reporting of outcomes
☒	☐	Estimates of effect sizes (e.g. Cohen's *d*, Pearson's *r*), indicating how they were calculated

Our web collection on statistics for biologists contains articles on many of the points above.

Software and code

Policy information about availability of computer code

Data collection	Thermo Tune 2.13
Data analysis	Xcalibur 4.2, Unidec 5.01 were used to open and assist data analysis, home made python script to process and fit data, scritps are available on Zenodo. Python 3.12.1, package versions: numpy (2.2.2), pandas (2.2.3), matplotlib.pyplot (3.10.0), lmfit (1.3.2).

For manuscripts utilizing custom algorithms or software that are central to the research but not yet described in published literature, software must be made available to editors and reviewers. We strongly encourage code deposition in a community repository (e.g. GitHub). See the Nature Portfolio guidelines for submitting code & software for further information.

Data

Policy information about availability of data

All manuscripts must include a data availability statement. This statement should provide the following information, where applicable:

- Accession codes, unique identifiers, or web links for publicly available datasets
- A description of any restrictions on data availability
- For clinical datasets or third party data, please ensure that the statement adheres to our policy

All the data required to evaluate the conclusions can be found in the main text or supplementary materials. Raw data has been uploaded to PRIDE (ID:PXD049009) . Scan extract and Python code is uploaded to Zenodo as stated in data availability statement.

Research involving human participants, their data, or biological material

Policy information about studies with human participants or human data. See also policy information about sex, gender (identity/presentation), and sexual orientation and race, ethnicity and racism.

Reporting on sex and gender	NA
Reporting on race, ethnicity, or other socially relevant groupings	NA
Population characteristics	NA
Recruitment	NA
Ethics oversight	NA

Note that full information on the approval of the study protocol must also be provided in the manuscript.

Field-specific reporting

Please select the one below that is the best fit for your research. If you are not sure, read the appropriate sections before making your selection.

☒ Life sciences ☐ Behavioural & social sciences ☐ Ecological, evolutionary & environmental sciences

For a reference copy of the document with all sections, see nature.com/documents/nr-reporting-summary-flat.pdf

Life sciences study design

All studies must disclose on these points even when the disclosure is negative.

Sample size	The size of the sample results from the replicates and the repeatability of the measurements in general. In total, the time-resolved measurements of SARS-CoV-2 His-tagged were carried out on three different days performing at least triplicate measurements.<br>The time-resolved measurements of tag-free polyprotein were carried out in at least triplicate measurements repeated on different days as following: SARS-CoV-2 4x3, SARS-CoV-1 3x3, MERS-CoV 2x3 and HCoV-229E 1x3. First number indicates repetition on different days, second number indicates technical replicates.<br>Interaction studies of nsp7-11 and nsp16 were repeated twice for each respective CoV strain and at least technical triplicates were taken
Data exclusions	data were excluded on discrete criteria, quality of capillary spray e.g. high spray variation, clogging of capillary, quality of buffer exchange and sample itself e.g. aggregation of sample, many salt adducts in mass spectra
Replication	protein production was conducted by two different persons; further, we performed experiments on different days and thus in the end probing several biological replicates and always recording technical replicates
Randomization	N/A
Blinding	our study does not comprise any group allocation upon data collection

Reporting for specific materials, systems and methods

We require information from authors about some types of materials, experimental systems and methods used in many studies. Here, indicate whether each material, system or method listed is relevant to your study. If you are not sure if a list item applies to your research, read the appropriate section before selecting a response.

Materials & experimental systems

n/a	Involved in the study	
☒	☐	Antibodies
☒	☐	Eukaryotic cell lines
☒	☐	Palaeontology and archaeology
☒	☐	Animals and other organisms
☒	☐	Clinical data
☒	☐	Dual use research of concern
☒	☐	Plants

Methods

n/a	Involved in the study	
☒	☐	ChIP-seq
☒	☐	Flow cytometry
☒	☐	MRI-based neuroimaging

Plants

Seed stocks	NA
Novel plant genotypes	NA
Authentication	NA
