# Supplementary figures and images for: Publisher Correction: The kinetics of nsp7-11 polyprotein processing and impact on complexation with nsp16 among human coronaviruses
Source: Nat Commun. 2025 Oct 9;16:8979. doi: 10.1038/s41467-025-64890-1 (PMC12511612; doi:10.1038/s41467-025-64890-1)

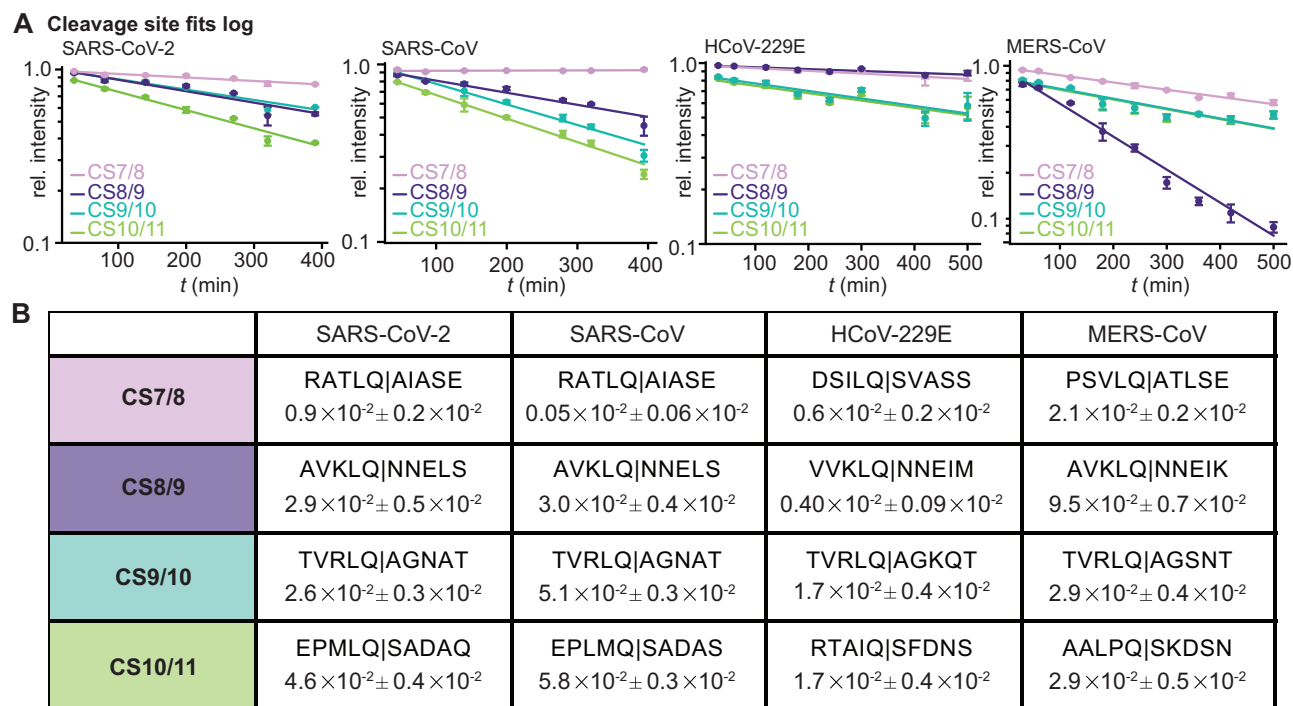

**Fig. 4 | Original, uncorrected.**

Supplement: Supplementary file 1 — Original, uncorrected Fig. 4. [file 41467_2025_64890_MOESM1_ESM.pdf]
